# Efficacy and safety of Tanshinone capsule in Acne vulgaris: a systematic review and meta-analysis

**DOI:** 10.3389/fphar.2025.1520039

**Published:** 2025-03-31

**Authors:** Yutong Deng, Ruli Feng, Bo Hu, Xuewen Ren, Fengchuan Zhang, Huishang Feng, Xuewan Wang, Yatong Li, Tangyunni Liu, Lingling Cai, Yuanwen Li

**Affiliations:** ^1^ Beijing University of Chinese Medicine, Beijing, China; ^2^ Department of Dermatology, Dongfang Hospital, Beijing University of Chinese Medicine, Beijing, China; ^3^ Department of Dermatology, Shanxi Traditional Chinese Medical Hospital, Shanxi, China; ^4^ Department of Dermatology, Dongzhimen Hospital, Beijing University of Chinese Medicine, Beijing, China

**Keywords:** Tanshinone capsule, Acne vulgaris, meta-analysis, systematic review, randomized controlled trial

## Abstract

**Objectives:**

To evaluate the efficacy and safety of Tanshinone capsule as a complementary therapy in managing of Acne Vulgaris.

**Methods:**

A systematic search of six databases was conducted to identify relevant randomized controlled trials (RCTs) from each database for nearly 20 years (from 1 Jan 2004, to 1 June 2024). The Cochrane Handbook was used to evaluate the risk of bias. Meta-analysis was performed using Review Manager 5.4.1, and publication bias was assessed the Stata SE 12.0 software. GRADEpro was used to assess the quality of the evidence.

**Results:**

A total of 2,969 participants from 28 studies were included. We found that Tanshinone capsules can reduce acne recurrence rates [risk ratio (*RR*) 0.44, 95% confidence interval (CI): 0.34 to 0.57, *p* < 0.00001]; downregulate levels of necrosis factor-alpha (TNF-α) [ mean difference (*MD*) 0.44, −10.18, 95% CI: −13.57 to −8.04, *p* < 0.00001], interleukin (IL) 4 (*MD* -6.46, 95%CI: −7.14 to −5.77, *p* < 0.00001), IL-6 (*MD* -16.14, 95%CI: −30.10 to −2.18, *p* = 0.02), IL-8 (*MD* -4.48, 95%CI: −8.30 to −0.65, *p* = 0.02) and testosterone (*MD* -14.50, 95%CI: −17.59 to −11.40, *p* < 0.00001); lower Global Acne Grading System (GAGS) score (*MD* -4.71, 95%CI: −7.62 to −1.80, *p* = 0.002); decrease sebum secretion rates (*MD* -0.29, 95%CI: −0.49 to −0.10, *p* = 0.003), but the regulation of Luteinizing hormone (LH), Follicle-stimulating hormone (FSH), Estradiol (E_2_) is not obvious. In terms of safety, the incidence of adverse events in the experimental group was less than that in the control group (*RR* 0.70, 95%CI: 0.56 to 0.87, *p* = 0.001). The Begg test and Egger test results indicated no publication bias. Furthermore, the levels of evidence ranged from very low to moderate due to risk of bias and heterogeneity.

**Conclusion:**

Tanshinone capsules can relieve the symptoms of acne vulgaris, regulate inflammatory cytokines and hormone levels in patients, and reduce recurrence. However, due to the limitations of this study, more multi-center and large-sample studies are needed to confirm these conclusions.

**Systematic Review Registration:**

https://www.crd.york.ac.uk/prospero/display_record.php?ID=CRD42024562320, identifier CRD42024562320.

## 1 Introduction

Acne vulgaris is a inflammatory dermatological disease that commonly occurs in areas rich in sebaceous glands, such as the face, chest, and back. The characteristic clinical features of acne include comedones, papules, pustules, nodules, cysts and scarring (Williams et al., 2012; Oon et al., 2019). The Global Burden of Disease Project estimates that acne affects approximately 9.4% of the global population, ranking it as the 8th most prevalent disease worldwide (Vos et al., 2012; Hay et al., 2014). Besides skin lesions, acne vulgaris can also cause a severe psychological burden and be associated with metabolic comorbidities, significantly affecting patients’ quality of life while increasing both individual and societal burdens (Stamu-O'Brien et al., 2021; Wang et al., 2022).

The current management of acne vulgaris is based on acne severity assessment and laboratory testing (Reynolds et al., 2024). Common treatments include topical retinoids, benzoyl peroxide, antibiotics, isotretinoin, contraceptives, and physical modalities, etc (Oon et al., 2019; Reynolds et al., 2024). However, increasing antibiotic resistance of *Cutibacterium acnes* (*C. acnes*) and the potential risk for adverse reactions remain significant challenges (Fox et al., 2016; Habeshian and Cohen, 2020). Therefore, complementary and alternative medicines (CAM) for acne treatment may be essential.

Tanshinone capsule is a traditional Chinese patent medicine made from the ethanol extract of *Danshen* (dried roots and rhizomes of *Salvia miltiorrhiza*), approved by the Chinese State Food and Drug Administration, the main active ingredients are tanshinone ⅡA (Tan ⅡA) and cryptotanshinone (CPT). The traditional Chinese medicine properties and therapeutic effects of *Danshen* are provided in Supplementary Table S3. According to Chinese pharmacopoeia standards, each capsule (0.25 g) contains no less than 16 mg of Tan ⅡA and 12 mg of CPT (State Food and Drug Administration of China, 2010). *In vitro* studies show that CPT and Tan ⅡA have antibacterial activity against *C. acnes*, *Staphylococcus epidermidis* and *Staphylococcus aureus*, which are acne-related pathogenic microorganisms (Zhu et al., 2022; Li and Zhou, 2018). Tan Ⅱa can inhibit the expression of toll-like receptor 2 (TLR2), nuclear factor-kappa B (NF-κB), and intercellular cell adhesion molecule-1 (ICAM-1), thereby suppressing *C. acnes*-induced inflammation and reducing the levels of inflammatory cytokines such as interleukin-1 beta (IL-1β), IL-8, and tumor necrosis factor-alpha (TNF-α) (Li and Zhou, 2018). CPT treatment alleviate acne inflammation, improve follicular keratinization and regulate the expression of IL-1α and androgen receptors (AR), demonstrating strong anti-inflammatory and anti-androgenic activities (Zuo et al., 2016).

In recent years, numerous clinical studies have explored the use of Tanshinone capsules for acne vulgaris. Therefore, we conducted a meta-analysis and systematic review of the past 2 decades to assess their efficacy and safety as a complementary therapy, providing evidence to guide clinical practice.

## 2 Methods

### 2.1 Study registration and ethics statements

The methods employed in this study were registered in PROSPERO (registration number: CRD42024562320), and strictly adhered to the PRISMA statement (Page et al., 2021).

### 2.2 Literature search

We performed a comprehensive search across 6 databases, including PubMed, the Cochrane Library, Embase, the Chinese Biomedical Literature Database (CBM), China National Knowledge Infrastructure (CNKI), Wanfang Databases (WF) and VIP, with no language restrictions, covering the 20-year period from 1 Jan 2004, to 1 June 2024. Two independent reviewers (DYT and FRL) conducted the search process. The search strategy integrated both Medical Subject Headings (MeSH) terms and free-text words. The language restriction was set to English and Chinese. The search terms included “acne vulgaris,” “acne,” and “Tanshinone capsule,” and related terms; the Chinese subject terms “Cuochuang” and “Danshentong” and related terms were used. The search strategies were adjusted according to the characteristics of different databases. Details of the search strategies are shown in Supplementary Materiale S4.

### 2.3 Inclusion criteria

#### 2.3.1 Types of studies

This study focused on randomized controlled trials (RCTs).

#### 2.3.2 Types of participants

Patients with Acne vulgaris diagnosed by a clinician or according to recognized diagnostic criteria. There were no restrictions on age, gender, race, or disease duration.

#### 2.3.3 Types of interventions

The control group received conventional therapies for acne vulgaris, including topical retinoids, topical antimicrobial therapy, oral antibiotics, oral isotretinoin, Hormonal therapy, other treatments and adjunctive therapies, etc. The experimental group received Tanshinone capsules used alone or in combination with the control group treatment.

#### 2.3.4 Outcome measures

The relapse rate after treatment was set as primary outcome, and the levels of inflammatory factors, hormone levels, sebum secretion rate and Global Acne Grading System (GAGS) score were set as secondary outcomes. The adverse events (AEs) was set as safety outcome.

### 2.4 Exclusion criteria

Studies meeting any of the following criteria were excluded: (1) studies focusing on other diseases accompanied by acneiform lesions, such as rosacea, SAPHO syndrome, polycystic ovary syndrome. (2) the treatment in control group or experiment group included unconventional therapy, such as acupuncture and moxibustion. (3) studies lacking mention of randomization (5) Studies lacking the specified outcome measures or reporting only AEs. (6) duplicate studies or those without full-text availability.

### 2.5 Literature screening and risk of bias

First, the primary literature retrieved from different databases was imported into the NoteExpress 3.8 software. After sequentially reading the titles, abstracts, and full texts, the final included studies were determined based on the inclusion and exclusion criteria. Second, two reviewers independently engaged in the screening of studies and extraction of data. Third, each included study was categorized, and Relevant data was extracted and recorded primarily including authors, publication years, sample sizes, gender ratios, intervention details, and outcome indicators. Fourth, the Cochrane Handbook (version 5.1.0) was used to evaluate the risk of bias according to the required items. Two independent reviewers (DYT and FRL) performed these tasks and if any discrepancies arose, they were resolved by a third researcher (LYW).

### 2.6 Data analysis and GRADE assessment

Meta-analysis was conducted using Review Manager (version 5.4). Continuous variables were expressed as mean difference (MD),while binary variables were expressed as risk ratio (RR), both with 95% confidence intervals (CIs). Heterogeneity was assessed using the *I* ^*2*^ test. A fixed-effects model was adopted when *I* ^*2*^ was <50%, indicating a low heterogeneity. Otherwise, a random-effects model was used. Subgroup analysis was conducted to investigate the potential influence of treatment duration and intervention methods for the experimental groups. Sensitivity analysis was conducted by excluding individual studies to assess the stability of the results. Publication bias was evaluated using Begg test and Egger test, facilitated by Stata SE 12.0 software. The quality of evidence for the outcome indicators was assessed using the GRADEpro system, which includes 5 downgrade factors and 3 upgrade factors.

## 3 Result

### 3.1 Literature screening

**FIGURE 1:**
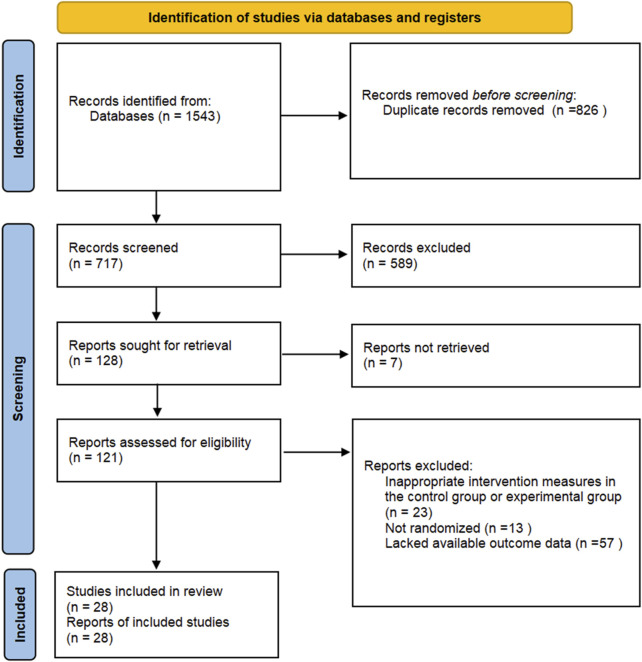
Literature selection process.

Initially, we retrieved and identified 1,523 articles that met the study period criteria, and then 822 duplicate studies were removed. After screening the titles and abstracts, 573 articles were excluded. Following a detailed evaluation of the full texts, an additional 100 studies were excluded (reasons for exclusion are shown in Supplementary Table S2). Consequently, a total of 28 studies (Chen and Li, 2023; Ma, 2023; Gu et al., 2023; Zhang et al., 2022; Zou, 2022; Pei and Shang, 2022; Zhang et al., 2021; Luo and Liu, 2021; Cai et al., 2021; Song, 2020; Lan, 2019; Kang and Yang, 2019; Kang et al., 2019; Liu et al., 2018; Yang T. et al., 2018; Xia, 2018; Wu, 2018; Peng, 2017; Zhou et al., 2017; Yan and Dong, 2016; Chen et al., 2015; Qin et al., 2015; He et al., 2015; Jiang and Sheng, 2015; Zhao and Yan, 2015; Lin et al., 2009; Chen and Liu, 2007; Cong et al., 2019) were included in the analysis. Details of the screening process are shown in Figure 1.

#### 3.2 Characteristics of included studies

The analysis included 28 studies with a total of 2,969 participants with acne vulgaris. There was a total of 1,599 participants in the experimental group and 1,370 participants in the control group. The age of participants ranged from 14 to 65 years old. The duration of acne ranged from 0.5 to 144 months. All trials were conducted in China and published in Chinese from 2007 to 2023. Among the 28 studies, 3 studies (Zhou et al., 2017; Lin et al., 2009; Chen and Liu, 2007) had experimental groups that received only Tanshinone capsules (*Hebei xinglong Xili Pharmaceutical Co., Ltd*) treatment, twenty one studies (Liu and Ma, 2023; Chen and Li, 2023; Ma, 2023; Gu et al., 2023; Zhang et al., 2022; Zou, 2022; Pei and Shang, 2022; Zhang et al., 2021; Luo and Liu, 2021; Cai et al., 2021; Song, 2020; Lan, 2019; Kang and Yang, 2019; Kang et al., 2019; Liu et al., 2018; Yang T. et al., 2018; Xia, 2018; Wu, 2018; Peng, 2017; Yan and Dong, 2016; Zhao and Yan, 2015) had treatment groups that received Tanshinone capsules treatment in addition to the control group’s treatment, and the remaining 4 studies (Chen et al., 2015; Qin et al., 2015; He et al., 2015; Jiang and Sheng, 2015) were three-arm studies that included both of these scenarios. The control group received one or more conventional treatments for acne vulgaris, including topical retinoids, topical antimicrobial therapy, oral antibiotics, oral isotretinoin, hormonal therapy, chemical peels and physical treatments. The characteristics of the included trials are shown in Table 1.

**TABLE 1:** Characteristics of the included trials.

Study ID	Sample size (Female %) T/C	Intervention		Treatment duration	Outcome
		T	C		
Liu and Ma (2023)	50(40)/50(38)	Tanshinone capsules 1g tid + OA + OR	OA + OR	1 m	②⑥
Chen and Li (2023)	43(58)/42(55)	Tanshinone capsules 1g tid + OR	OR	6w	②⑥
Ma (2023)	43(42)/43(49)	Tanshinone capsules 1g tid + OR	OR	6w	①②⑥
Gu et al. (2023)	45(53)/45(58)	Tanshinone capsules 1g tid + OR	OR	8w	②⑤⑥
Zou (2022)	50(44)/50(48)	Tanshinone capsules 1g tid + OR	OR	8w	①⑥
Pei and Shang (2022)	20(50)/20(45)	Tanshinone capsules 1g tid + TA + PT	TA + PT	4w	④⑥
Zhang et al. (2021)	52(54)/52(48)	Tanshinone capsules 1g tid + PT	PT	3 m	①⑥
Luo and Liu (2021)	69(49)/68(44)	Tanshinone capsules 1g tid + OA + OR	OA + OR	1 m	②⑥
Cai et al. (2021)	41(46)/39(49)	Tanshinone capsules 1g tid + OA	OA	4w	②
Song (2020)	44(57)/44(52)	Tanshinone capsules 1g tid + TR + TA + PT	TR + TA + PT	8w	②
Lan (2019)	89(44)/89(43)	Tanshinone capsules 1g tid + OR	OR	6w	①⑥
Kang and Yang (2019)	60(53)/60(58)	Tanshinone capsules 1g tid + OA + TA	OA + TA	12w	①⑥
Kang et al. (2019)	30(47)/30(40)	Tanshinone capsules 1g tid + OR	OR	6w	①⑥
Cai et al. (2021)	41(46)/39(49)	Tanshinone capsules 1g tid + OA	OA	4w	②
Liu et al. (2018)	25(44)/26(46)	Tanshinone capsules 1g tid + PT	PT	2 m	②③⑥
Yang et al. (2018a)	44(41)/44(43)	Tanshinone capsules 1g tid + OA	OA	6w	①②⑥
Xia (2018)	40(53)/40(55)	Tanshinone capsules 1g tid + CP	CP	NR	③
Wu (2018)	60(38)/60(40)	Tanshinone capsules 1-0.75g tid + TR + TA	TR + TA	8w	②
Peng (2017)	60(58)/60(63)	Tanshinone capsules 1g tid + OA	OA	8w	③⑤
Zhou et al. (2017)	31(100)/31(100)	Tanshinone capsules 0.75g tid	HT	4w	①②⑥
Yan and Dong (2016)	52(NR)/50(NR)	Tanshinone capsules 1g tid + OR	OR	6w	①⑥
Chen et al. (2015)	82(100)/83(100)	Tanshinone capsules 1g tid	HT	6w	⑤⑥
Chen et al. (2015)	86(100)/83(100)	Tanshinone capsules 1g tid + HT	HT	6w	⑤⑥
Qin et al. (2015)	32(NR)/32(NR)	Tanshinone capsules 1g tid	TA	8w	①⑥
Qin et al. (2015)	34(NR)/32(NR)	Tanshinone capsules 1g tid + TA	TA	8w	①⑥
He et al. (2015)	52(46)/54(39)	Tanshinone capsules 1g qid	CP	8w	①⑥
He et al. (2015)	55(44)/54(39)	Tanshinone capsules 1g qid + CP	CP	8w	①⑥
Jiang and Sheng (2015)	30(40)/30(47)	Tanshinone capsules 1g tid	OR	8w	②⑥
Jiang and Sheng (2015)	30(43)/30(47)	Tanshinone capsules 1g tid + OA	OR	8w	②⑥
Zhao and Yan (2015)	30(53)/30(60)	Tanshinone capsules 1g tid + TA + PT	TA + PT	8w	②
Lin et al. (2009)	42(40)/34(41)	Tanshinone capsules 1g tid	OA	6w	①⑥
Chen and Liu (2007)	85(47)/71(51)	Tanshinone capsules 1-0.75g tid	OA	8w	①⑥

T, treatment group; C, control group; tid, thrice daily; qid, four times daily; w,week; m, month; TA, topical antimicrobial; TR, topical retinoids; OA, oral antibiotics; OI, oral isotretinoin; CP, chemical peels; HT, hormonal therapy; PT, physical treatment; NR: not report; ① Relapse rate; ② Inflammatory factors levels; ③ Sebum secretion rate; ④ GAGS, scores; ⑤ Hormone levels; ⑥ AEs.

### 3.3 Risk of bias assessment

**FIGURE 2:**
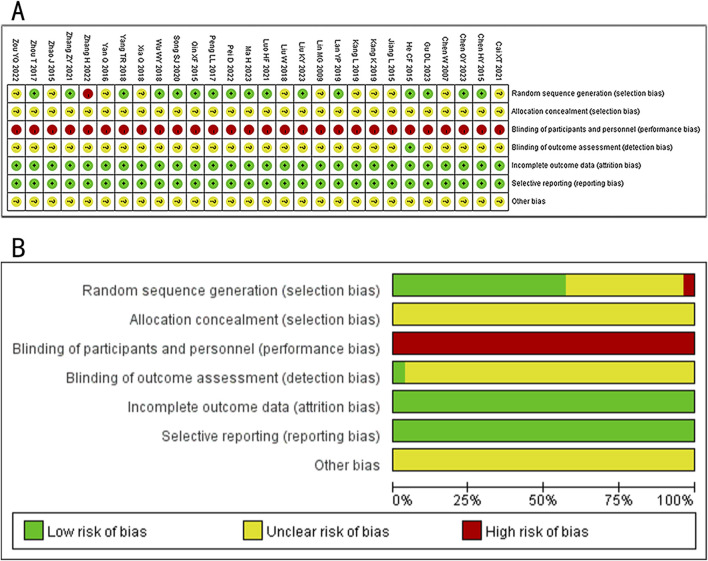
Risk of bias. **(A)** Risk of bias graph. **(B)** Risk of bias summary.

Regarding selection bias, fifteen studies (Liu and Ma, 2023; Chen and Li, 2023; Ma, 2023; Pei and Shang, 2022; Zhang et al., 2021; Luo and Liu, 2021; Song, 2020; Lan, 2019; Yang T. et al., 2018; Wu, 2018; Peng, 2017; Zhou et al., 2017; Chen et al., 2015; Qin et al., 2015; He et al., 2015) used the random number table method and 1 study (Gu et al., 2023) used random drawing method, which was rated as low. One study (Zhang et al., 2022) was considered high risk due to random grouping based on the order of patient visits. The remaining 11 studies (Zou, 2022; Cai et al., 2021; Kang and Yang, 2019; Kang et al., 2019; Liu et al., 2018; Xia, 2018; Yan and Dong, 2016; Jiang and Sheng, 2015; Zhao and Yan, 2015; Lin et al., 2009; Chen and Liu, 2007) did not report the method for generating random sequences and were rated as unclear risk. None of the studies explained the randomization method in detail, which was considered to be an unclear risk of bias. Due to the significant differences in the formulation of treatments between the treatment and control groups, performance bias was rated as high. For detection bias, one study (He et al., 2015) was considered low risk as outcome data collection and assessment was conducted under blinding. All studies had no patients fell off, all studies reported test indicators as planned, and there was no selective reporting of research results. It is unclear whether there were other examples of bias. The Risk of bias graph is shown in Figure 2.

### 3.4 Effectiveness and safety of Tanshinone capsules

#### 3.4.1 Primary outcome

**FIGURE 3:**
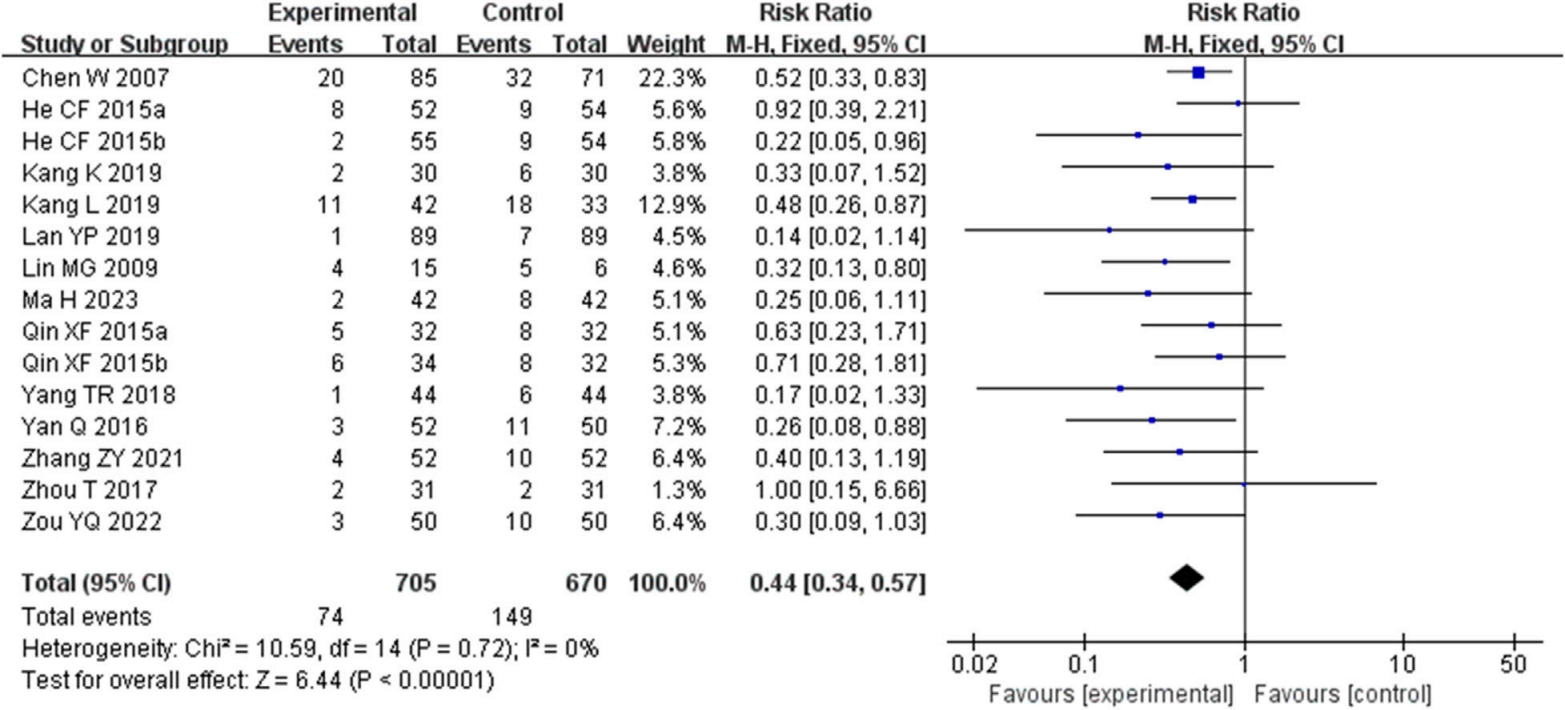
Forest plot for the total relapse rate of Tanshinone capsules versus Control group.

A total of 15 studies (including two three-arm studies) (Ma, 2023; Zou, 2022; Zhang et al., 2021; Lan, 2019; Kang and Yang, 2019; Kang et al., 2019; Yang T. et al., 2018; Zhou et al., 2017; Yan and Dong, 2016; Qin et al., 2015; He et al., 2015; Lin et al., 2009; Chen and Liu, 2007) evaluated the relapse rate, involving 1,289 patients. The fixed-effects model was used for subsequent meta-analysis because of the low heterogeneity among the studies (*p* = 0.72, *I* ^*2*^ = 0%). We found that the recurrence rate in the experimental group was lower than the control group (RR = 0.44, 95%CI 0.34 to 0.57, *p* < 0.00001) (Figure 3). The sensitivity analysis demonstrated low sensitivity, indicating that the results were robust against the exclusion of any single study (Supplementary Figure S1A). Although Chen and Liu (2007) contributed a heavy weight (22.3%) to the analysis, the sensitivity analysis results remained consistent after its exclusion (RR _exclusion Chen W 2007_ = 0.42, 95% CI: 0.31 to 0.56, *p* < 0.00001, *I* ^*2*^ = 0).

**FIGURE 4:**
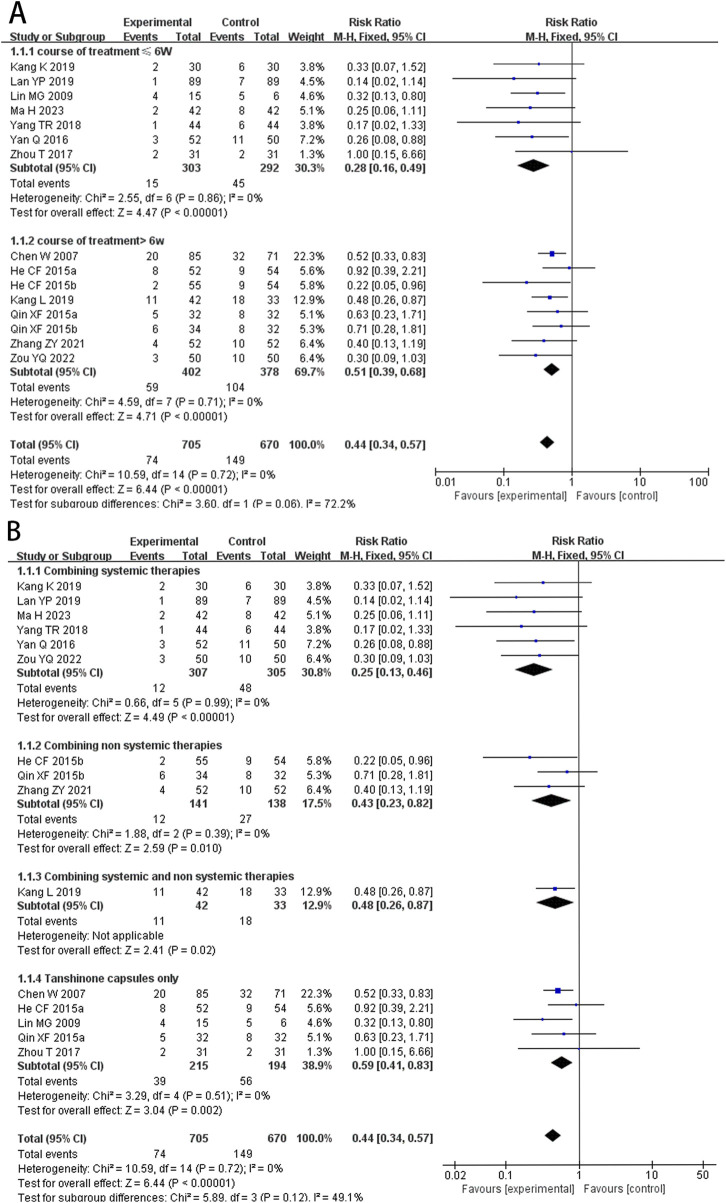
Forest plot for subgroup analysis of the relapse rate. **(A)** Different treatment course. **(B)** Different intervention methods for the experimental groups.

At the same time, to avoid the influence of different intervention methods on the analysis results, we conducted a subgroup analysis based on treatment durations (≤6 weeks, >6 weeks) and different intervention methods for the experimental group (combining systemic therapies, combining non-systemic therapies, combining both systemic and non-systemic therapies, and Tanshinone capsules only). The results suggested that regardless of whether the treatment period exceeded 6 weeks the recurrence rate in the experimental group was lower than that in the control group (RR_≤ 6 weeks_ = 0.28, 95%CI: 0.16 to 0.49, *p* < 0.00001, *I* ^*2*^ = 0%; RR _>6 weeks_ = 0.51, 95%CI: 0.39 to 0.68, *p* < 0.00001, *I* ^*2*^ = 0%) (Figure 4A). Additionally the results of the different intervention methods subgroup analysis were consistent with the overall results (RR _Combining systemic therapies_ = 0.25, 95%CI: 0.13 to 0.46, *p* < 0.00001; RR _Combining non systemic therapies_ = 0.43, 95%CI: 0.23 to 0.82, *p* = 0.01; RR _Combining systemic and non systemic therapies_ = 0.48, 95%CI: 0.26 to 0.87, *p* = 0.02; RR _Tanshinone capsules only_ = 0.59, 95%CI: 0.41 to 0.83, *p* = 0.002) (Figure 4B).

#### 3.4.2 Secondary outcomes

##### 3.4.2.1 Levels of inflammatory factors

###### 3.4.2.1.1 TNF-α

**FIGURE 5:**
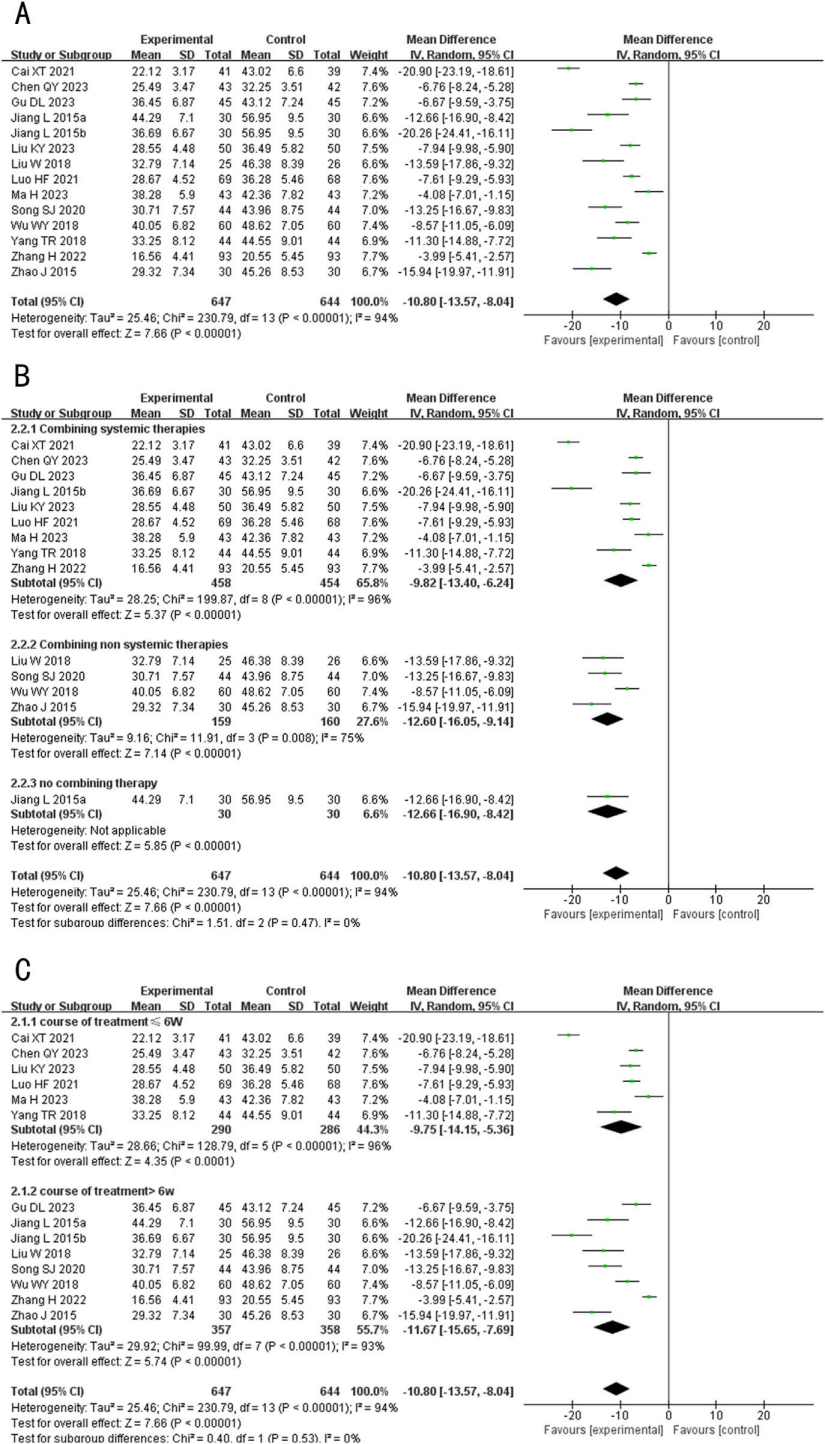
Forest plot of TNF-α level. **(A)** Forest plot for the total result of Tanshinone capsules versus Control group. **(B)** Subgroup analysis forest plot of different treatment course. **(C)** Subgroup analysis forest plot of different intervention methods for the experimental groups.

A total of 14 studies (including one three-arm study) (Liu and Ma, 2023; Chen and Li, 2023; Ma, 2023; Gu et al., 2023; Zhang et al., 2022; Luo and Liu, 2021; Cai et al., 2021; Song, 2020; Liu et al., 2018; Yang T. et al., 2018; Wu, 2018; Jiang and Sheng, 2015; Zhao and Yan, 2015) evaluated TNF-α levels, involving 1,261 patients. The random-effects model was used for subsequent meta-analysis because of the high heterogeneity among the studies (*p* < 0.00001, *I* ^*2*^ = 94%). We found that TNF-α levels in the experimental group were lower than in the control group (MD = −10.18, 95%CI: −13.57 to −8.04, *p* < 0.00001) (Figure 5A). Sensitivity analysis demonstrated low sensitivity, indicating that the results were robust against the exclusion of any single study (Supplementary Figure S1B).

Subgroup analysis showed that regardless of treatment duration or intervention method, TNF-α levels in the treatment group were consistently lower than in the control group, aligning with the overall results (MD _≤6 weeks_ = −9.75, 95%CI: −14.15 to −5.36, *p* < 0.00001; MD _>6 weeks_ = −11.67, 95%CI: −15.65 to −7.69, *p* < 0.00001; MD _Combining systemic therapies_ = −9.82, 95%CI: −13.40 to −6.24, *p* < 0.00001; MD _Combining non systemic therapies_ = −12.60, 95%CI: −16.05 to −9.14, *p* < 0.00001; MD _Tanshinone capsules only_ = −10.80, 95%CI: −13.57 to −8.04, *p* < 0.00001) (Figures 5B,C).

###### 3.4.2.1.2 IL-4

**FIGURE 6:**
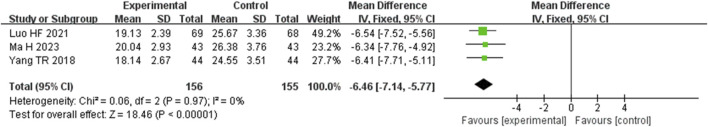
Forest plot for the IL-4 of Tanshinone capsules versus Control group.

A total of 3 studies (Ma, 2023; Luo and Liu, 2021; Yang T. et al., 2018) evaluated the IL-4 levels, involving 311 patients. The fixed-effects model was used for subsequent meta-analysis because of the low heterogeneity among the studies (*p* = 0.97, *I* ^*2*^ = 0%). The results indicated that after treatment, the level of IL-4 in the experimental group was lower than in the control group after treatment (MD = −6.46, 95%CI: −7.14 to −5.77, *p* < 0.00001) (Figure 6). And the sensitivity analysis demonstrated low sensitivity, indicating that the results were robust against the exclusion of any single study (Supplementary Figure S1C). Since all three studies had treatment durations of less than 6 weeks and the treatment groups combined systemic therapies, subgroup analysis was not conducted.

###### 3.4.2.1.3 IL-6

**FIGURE 7:**
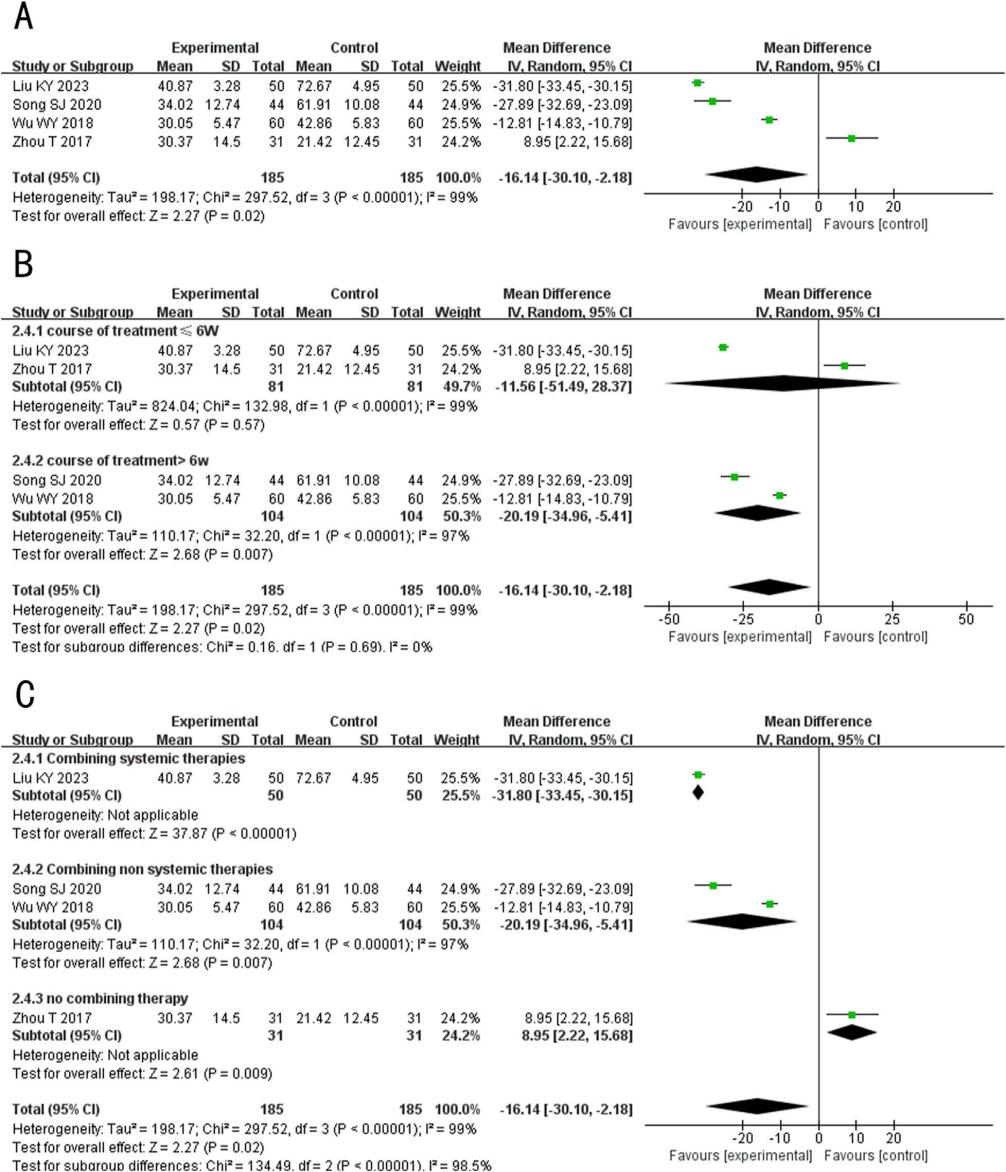
Forest plot of IL-6 level. **(A)** Forest plot for the total result of Tanshinone capsules versus Control group. **(B)** Subgroup analysis forest plot of different treatment course. **(C)** Subgroup analysis forest plot of different intervention methods for the experimental groups.

A total of 4 studies (Liu and Ma, 2023; Song, 2020; Wu, 2018; Zhou et al., 2017) evaluated the IL-6 levels, involving 370 patients. The random-effects model was used for subsequent meta-analysis because of the high heterogeneity among the studies (*p* < 0.00001, *I* ^*2*^ = 99%). The results showed that after treatment, the level of IL-6 in the experimental group was lower than the control group (MD = −16.14, 95%CI: −30.10 to −2.18, *p* = 0.02) (Figure 7A). In the sensitivity analysis, no single study remarkably affected the effect sizes of IL-6 levels. However, after removing the studies Liu and Ma (2023), Song (2020), Wu (2018), the result was no longer significant (MD _exclusion Liu KY 2023_ = −10.18, 95%CI: −26.24 to 4.64, *p* = 0.17; MD _exclusion Song SJ 2020_ = −12.21, 95%CI: −29.63 to 5.22, *p* = 0.17; MD _exclusion Wu WY 2018_ = −17.16, 95%CI: −36.82 to 2.49, *p* = 0.09) (Supplementary Figure S1D).

Subgroup analysis results showed that When the treatment duration was ≤6 weeks, there was no significant difference in the level of IL-6 between the two groups (MD≤_6 weeks_ = −11.56, 95%CI: −51.49 to 28.37, *p* = 0.57). However, when the treatment duration exceeded 6 weeks, IL-6 levels in the experimental group were significantly lower (MD _>6 weeks_ = −20.19, 95%CI: −34.96 to −5.41, *p* = 0.007) (Figure 7B). In the subgroup analysis of different intervention methods, the results for “Combining systemic therapies” and “Combining non-systemic therapies” were consistent with the overall findings (MD _Combining systemic therapies_ = −31.80, 95%CI: −33.45 to −30.15, *p* < 0.00001; MD _Combining non systemic therapies_ = −20.19, 95%CI: −34.96 to −5.41, *p* < 0.00001). However, the “Tanshinone capsules only” subgroup showed opposite results compared to the overall findings (MD _Tanshinone capsules only_ = 8.95, 95%CI: 2.22 to −8.04, *p* < 0.00001) (Figure 7C).

###### 3.4.2.1.4 IL-8

**FIGURE 8:**
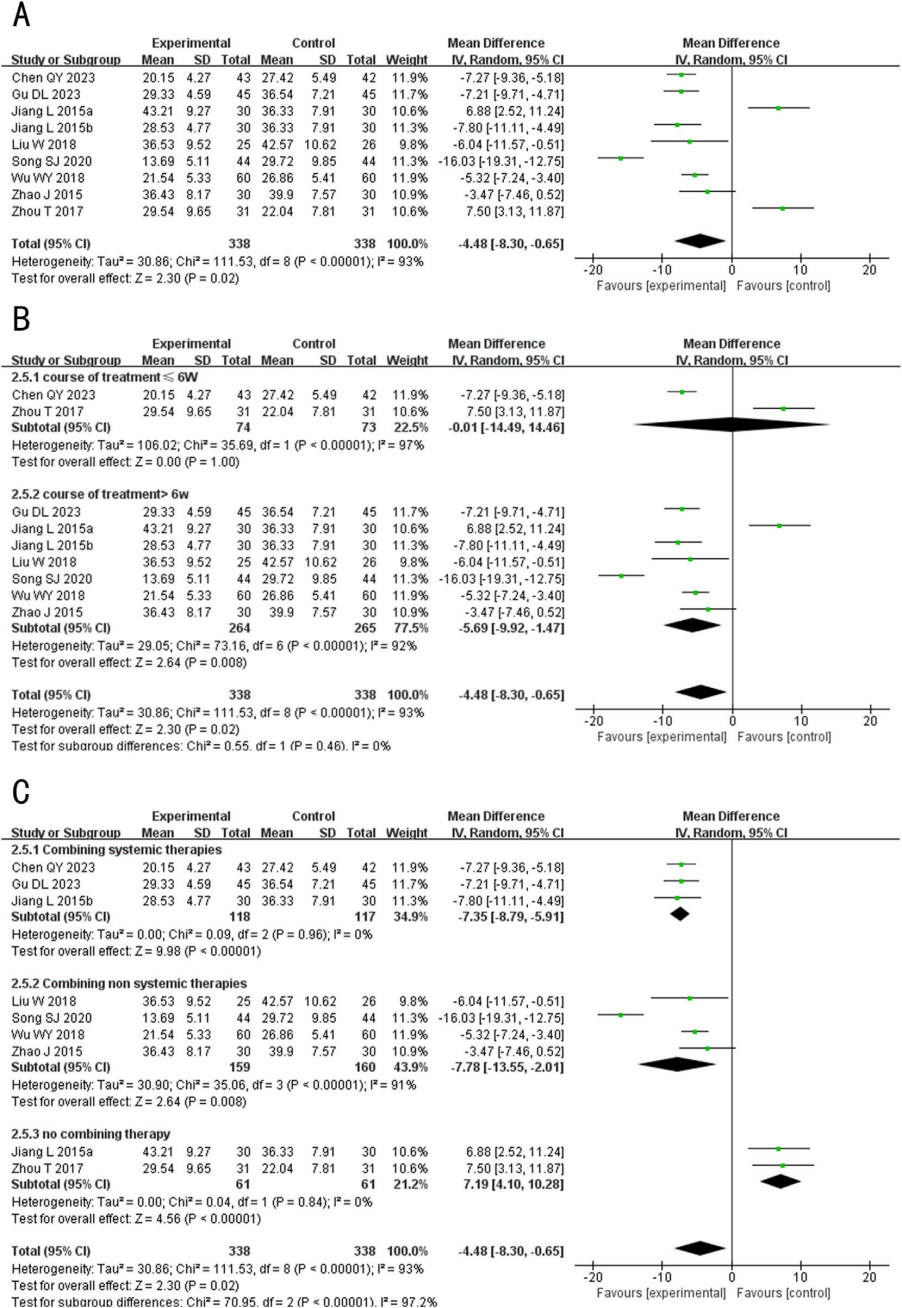
Forest plot of IL-8 level. **(A)** Forest plot for the total result of Tanshinone capsules versus Control group. **(B)** Subgroup analysis forest plot of different treatment course. **(C)** Subgroup analysis forest plot of different intervention methods for the experimental groups.

A total of 9 studies (including one three-arm study) (Chen and Li, 2023; Gu et al., 2023; Song, 2020; Liu et al., 2018; Wu, 2018; Zhou et al., 2017; Jiang and Sheng, 2015; Zhao and Yan, 2015) evaluated the IL-8 levels, involving 646 patients. The random-effects model was used for subsequent meta-analysis because of the high heterogeneity among the studies (*p* < 0.00001, *I* ^*2*^ = 93%). The results showed that after treatment, the IL-8 levels in the experimental group was lower than the control group (MD = −4.48, 95%CI: −8.30 to −0.65, *p* = 0.02) (Figure 8A). In the sensitivity analysis, no single study remarkably affected the effect sizes of IL-8 levels. However, after excluding the studies Chen and Li, (2023), Gu et al. (2023), Lan, (2019), Wu (2018), Jiang and Sheng (2015), the result was no longer significant (MD _exclusion Chen QY 2023_ = −4.06, 95%CI: −8.66 to 0.53, *p* = 0.08; MD _exclusion Gu DL 2023_ = −4.08, 95%CI: −8.54 to 0.37, *p* = 0.07; MD _exclusion Song SJ 2020_ = −3.07, 95%CI: −6.53 to 0.39, *p* = 0.08; MD _exclusion Wu WY 2018_ = −4.32, 95%CI: −9.03 to 0.40, *p* = 0.07; MD _exclusion Jiang L 2015b_ = −4.04, 95%CI: −8.31 to 0.24, *p* = 0.06) (Supplementary Figure S1E).

Subgroup analysis results showed that When the treatment duration was ≤6 weeks, there was no significant difference in the IL-8 levels between the two groups (MD≤_6 weeks_ = −0.01, 95%CI: −14.49 to 14.46, *p* = 1.00). However, when the treatment duration exceeded 6 weeks, IL-6 levels in the experimental group were significantly lower (MD _> 6 weeks_ = −5.69, 95%CI: −9.92 to −1.47, *p* = 0.008) (Figure 8B). In the subgroup analysis of different intervention methods, the results for “Combining systemic therapies” and “Combining non-systemic therapies” were consistent with the overall findings (MD _Combining systemic therapies_ = −7.35, 95%CI: −8.97 to −5.91, *p* < 0.00001; MD _Combining non systemic therapies_ = −7.78, 95%CI: −13.55 to −2.01, *p* = 0.008), while the “Tanshinone capsules only” subgroup showed opposite results compared to the overall findings (MD _Tanshinone capsules only_ = 7.19, 95%CI: 4.10 to 10.28, *p* < 0.00001) (Figure 8C).

##### 3.4.2.2 Sebum secretion rate

**FIGURE 9:**
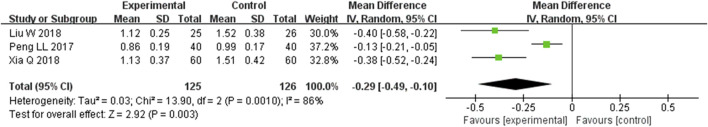
Forest plot for the sebum secretion rate of Tanshinone capsules versus Control group.

A total of 3 studies (Liu et al., 2018; Xia, 2018; Peng, 2017) evaluated the sebum secretion rate, involving 251 patients. The random-effects model was used for subsequent meta-analysis because of the high heterogeneity among the studies (*p* = 0.0010, *I* ^*2*^ = 86%). The results showed that after treatment, the sebum secretion rate in the experimental group was lower than the control group (MD = −0.29, 95%CI: −0.49 to −0.10, *p* = 0.003) (Figure 9). Sensitivity analysis revealed that heterogeneity significantly decreased (*p* = 0.86, *I* ^*2*^ = 0%) after excluding the study Peng LL 2017 (Peng, 2017), which had a lower initial sebum secretion rate and may have contributed to methodological heterogeneity (Supplementary Figure S1F).

##### 3.4.2.3 GAGS score

**FIGURE 10:**
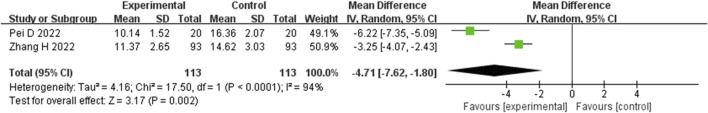
Forest plot for the GAGS scores of Tanshinone capsules versus Control group.

A total of 2 studies (Zhang et al., 2022; Pei and Shang, 2022) evaluated the GAGS score, involving 226 patients. The random-effects model was used for subsequent meta-analysis because of the high heterogeneity among the studies (*p <* 0.0001, *I* ^*2*^ = 94%). The results showed that after treatment, the GAGS score in the treatment group was lower than the control group (MD = −4.71, 95%CI: −7.62 to −1.80, *p* = 0.002) (Figure 10). Sensitivity analysis demonstrated low sensitivity, indicating that the results were robust against the exclusion of any single study (Supplementary Figure S1G).

##### 3.4.2.4 Levels of hormone

###### 3.4.2.4.1 Luteinizing hormone

**FIGURE 11:**
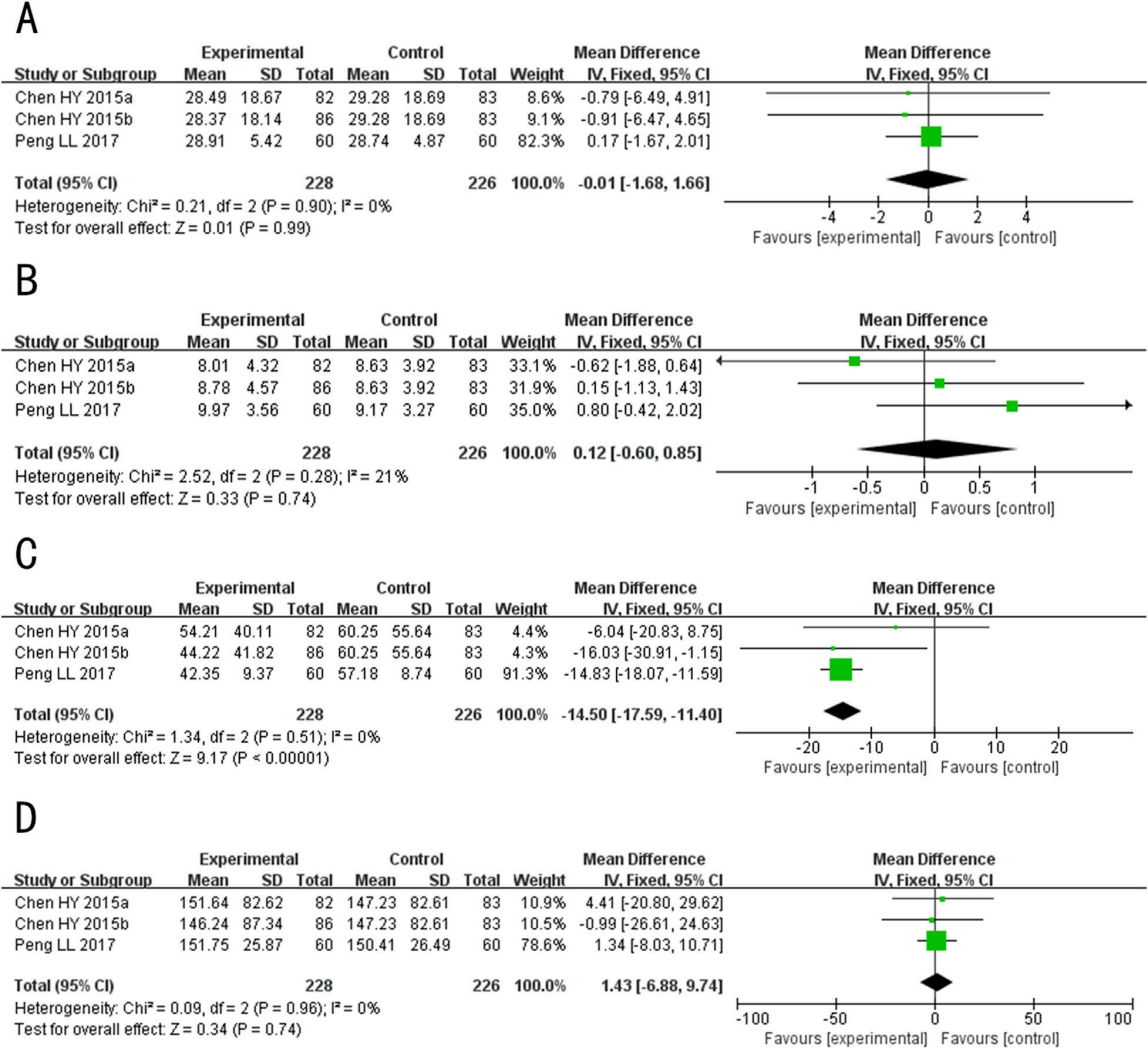
Forest plot for the hormone Levels of Tanshinone capsules versus Control group. **(A)** LH. **(B)** FSH. **(C)** T. **(D)** E_2_.

A total of 3 studies (including one three-arm study) (Peng, 2017; Chen et al., 2015) evaluated luteinizing hormone (LH) levels, involving 371 patients. The fixed-effects model was used for subsequent meta-analysis because of the low heterogeneity among the studies (*p* = 0.90, *I* ^*2*^ = 0%). The results showed no statistically significant difference in LH levels between the experimental and control groups after treatment (MD = −0.01, 95%CI: −1.68 to 1.66, *p* = 0.99) (Figure 11A). The sensitivity analysis demonstrated low sensitivity, indicating that the results were robust against the exclusion of any single study (Supplementary Figure S1H).

###### 3.4.2.4.2 Follicle-stimulating hormone

A total of 3 studies (including one three-arm study) (Peng, 2017; Chen et al., 2015) evaluated follicle-stimulating hormone (FSH) levels, involving 371 patients. The fixed-effects model was used for subsequent meta-analysis because of the low heterogeneity among the studies (*p* = 0.28, *I* ^*2*^ = 21%). The results showed no statistically significant difference in the level of FSH between the experimental and control groups after treatment (MD = 0.12, 95%CI: −0.60 to 0.85, *p* = 0.74) (Figure 11B). The sensitivity analysis demonstrated that no single study had a significant impact on the overall results and the effect sizes of the FSH levels after treatment. However, after excluding Chen HY 2015b (Chen et al., 2015), the heterogeneity increased (*p* = 0.11, *I* ^*2*^ = 60%) (Supplementary Figure S1I).

###### 3.4.2.4.3 Testosterone

A total of 4 studies (including one three-arm study) (Gu et al., 2023; Peng, 2017; Chen et al., 2015) evaluated the testosterone (T) levels, but the study Gu DL 2023 (Gu et al., 2023) employed a different detection method, resulting in significant variations in the values. Consequently, only 3 studies (Peng, 2017; Chen et al., 2015) were ultimately combined (including one three-arm study), involving 371 patients. The fixed-effects model was used for subsequent meta-analysis because of the low heterogeneity among the studies (*p* = 0.52, *I* ^*2*^ = 0%). The results showed that after treatment, T levels in the experimental group was lower than the control group (MD = −14.50, 95%CI: −17.59 to −11.40, *p* < 0.00001) (Figure 11C). In the sensitivity analysis, after excluding Peng LL 2017 (Peng, 2017), the result exceeded the original 95% confidence interval (MD _exclusion Peng LL 2017_ = −11.0, 95%CI: −21.49 to −0.25, *p* = 0.04) (Supplementary Figure S1J). This indicates that Peng LL 2017 (Peng, 2017) had a significant impact on the overall results and may be a key source of potential heterogeneity. Consequently, the meta-analysis results appear to be sensitive to this particular study, suggesting potential instability in the overall findings. We speculate that may arise from differences in patient demographics, as the majority of participants in other studies were female, potentially influencing the sensitivity analysis outcome.

###### 3.4.2.4.4 Estradiol

A total of 4 studies (including one three-arm study) (Gu et al., 2023; Peng, 2017; Chen et al., 2015) evaluated the Estradiol (E_2_) levels after treatment, but the study Gu DL 2023 (Gu et al., 2023) employed a different detection method, resulting in significant variations in reported values. Consequently, only 3 studies (Peng, 2017; Chen et al., 2015) were ultimately combined (including one three-arm study), involving 371 patients. The fixed-effects model was used for subsequent meta-analysis because of the low heterogeneity among the studies (*p* = 0.96, *I* ^*2*^ = 0%). The results indicated no statistically significant difference in E_2_ levels between the experimental and control groups after treatment (MD = 1.43, 95%CI: −6.88 to 9.74, *p* = 0.74) (Figure 11D). The sensitivity analysis demonstrated low sensitivity, indicating that the results were robust against the exclusion of any single study (Supplementary Figure S1K).

##### 3.4.2.5 Adverse events

**FIGURE 12:**
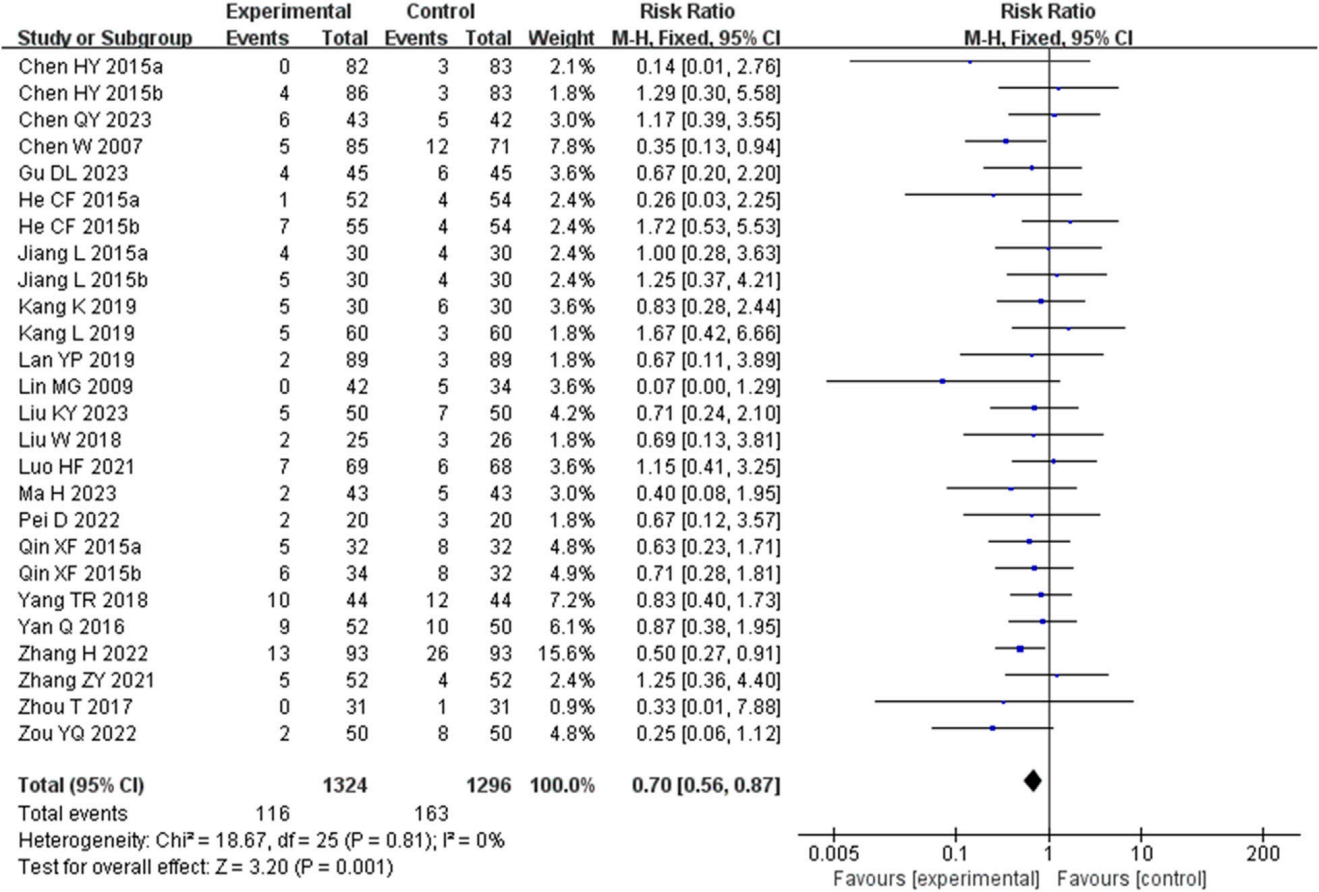
Forest plot for the AEs of Tanshinone capsules versus Control group.

A total of 27 studies (including four three-arm studies) (Liu and Ma, 2023; Chen and Li, 2023; Ma, 2023; Gu et al., 2023; Zhang et al., 2022; Zou, 2022; Pei and Shang, 2022; Zhang et al., 2021; Luo and Liu, 2021; Cai et al., 2021; Lan, 2019; Kang and Yang, 2019; Kang et al., 2019; Liu et al., 2018; Yang T. et al., 2018; Zhou et al., 2017; Yan and Dong, 2016; Chen et al., 2015; Qin et al., 2015; He et al., 2015; Jiang and Sheng, 2015; Lin et al., 2009; Chen and Liu, 2007) reported the occurrence of AEs during treatment. However, Cai XT 2021 (Cai et al., 2021) reported no observed adverse reactions in either the experimental or control group. Consequently, only 26 studies (including four three-arm studies) (Liu and Ma, 2023; Chen and Li, 2023; Ma, 2023; Gu et al., 2023; Zhang et al., 2022; Zou, 2022; Pei and Shang, 2022; Zhang et al., 2021; Luo and Liu, 2021; Lan, 2019; Kang and Yang, 2019; Kang et al., 2019; Liu et al., 2018; Yang T. et al., 2018; Zhou et al., 2017; Yan and Dong, 2016; Chen et al., 2015; Qin et al., 2015; He et al., 2015; Jiang and Sheng, 2015; Lin et al., 2009; Chen and Liu, 2007) were ultimately combined, involving 2,421 patients. Three studies (Yan and Dong, 2016; Qin et al., 2015) (including one three-arm study) did not provide details of AEs. The main adverse reactions observed included gastrointestinal discomfort, skin flushing, and itching, among others. The fixed-effects model was used for subsequent meta-analysis because of the low heterogeneity among the studies (*p* = 0.81, *I* ^*2*^ = 0%). The results indicated that the incidence of AEs during treatment was significantly lower in the experimental group compared to the control group (RR = 0.70, 95%CI: 0.56 to 0.87, *p* = 0.001) (Figure 12). The sensitivity analysis demonstrated low sensitivity, indicating that the results were robust against the exclusion of any single study (Supplementary Figure S1L).

### 3.5 Publication bias

**FIGURE 13:**
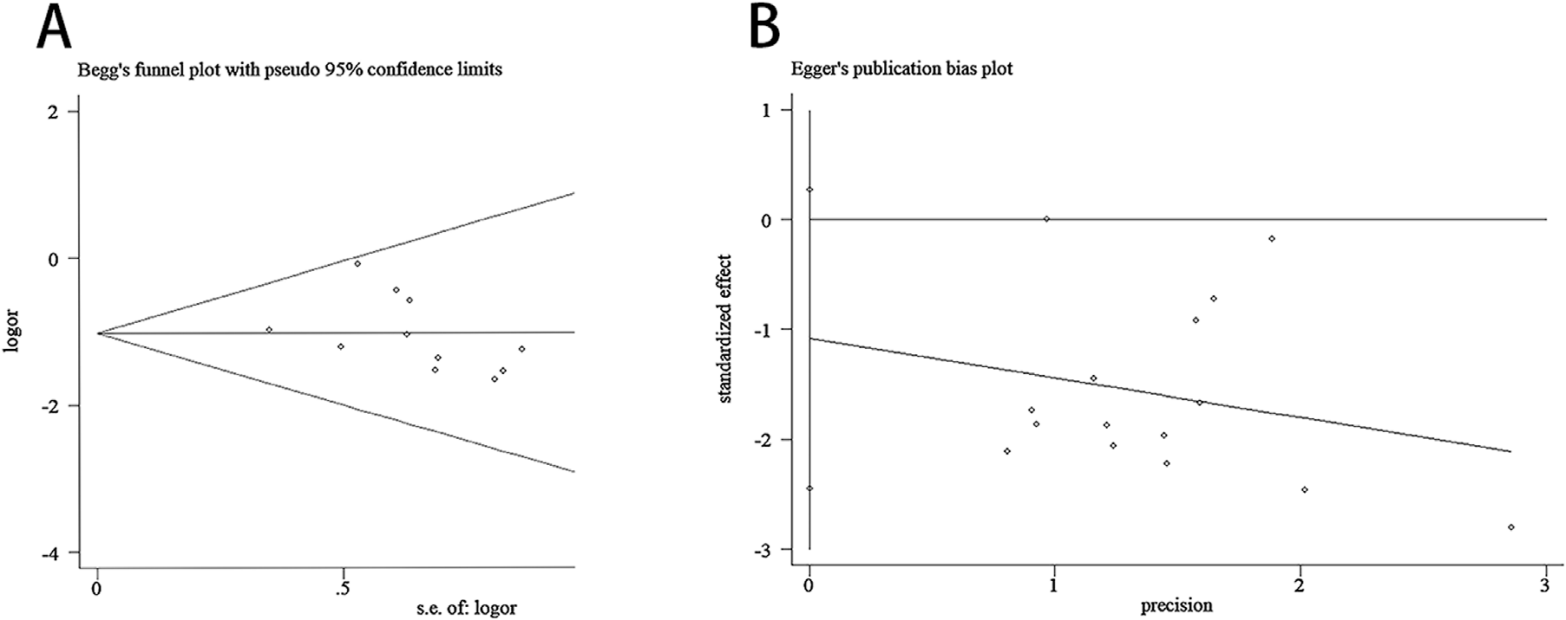
Begg test and Egger test. **(A)** Begg test for the relapse rate. **(B)** Egger test for the relapse rate.

Regarding the relapse rate, the Begg test results were: z = 2.47 (continuity corrected), Pr > |z| = 0.013 (continuity corrected), thus we conducted trim-and-fill test analysis, the results showed that Q = 1.961, p = 1.000, IOR = 0.416, 95%CI: 0.086 to 0.746; and the Egger test results were: t = −1.72, P > |t| = 0.108. These results indicated the absence of statistical significance in terms of publication bias (Figure 13).

### 3.6 Evidence quality assessment

**FIGURE 14:**
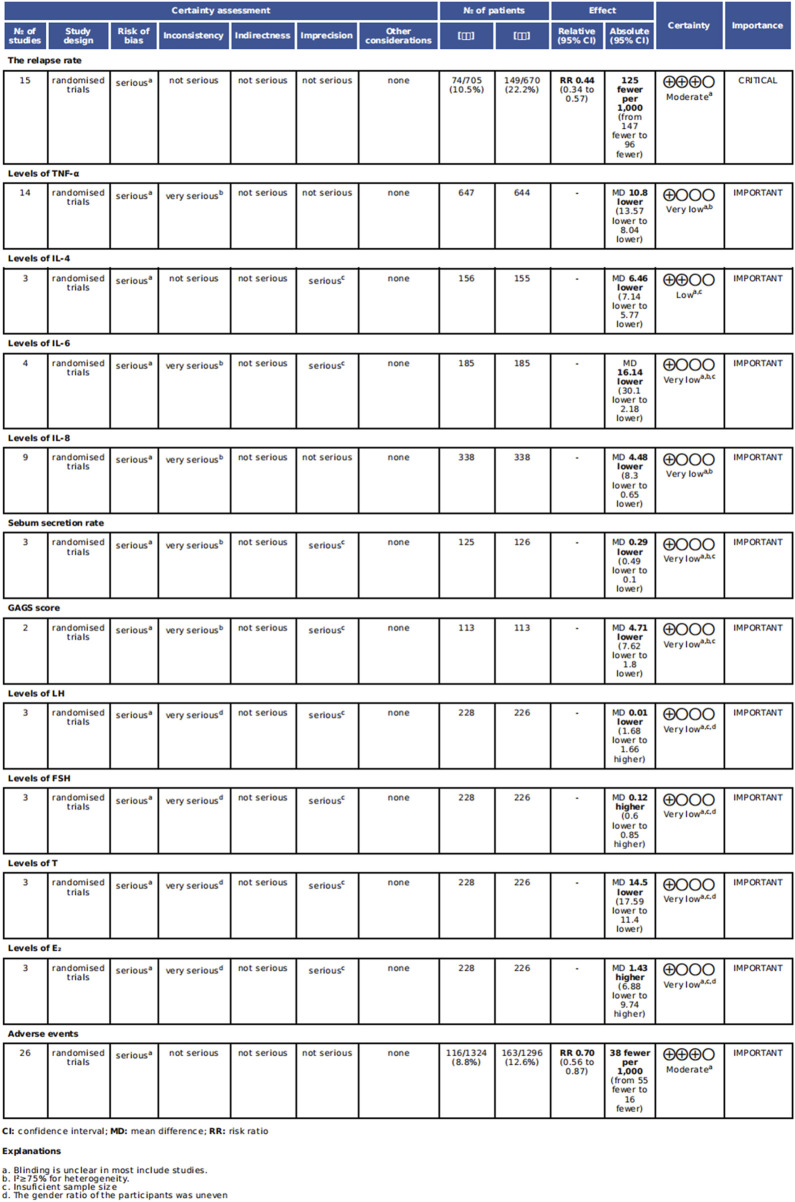
Grade evaluation of endpoint indicators.

The quality of evidence was assessed using the GRADEpro. The quality of evidence ranged from very low to moderate. The primary reasons for downgrading were inconsistency (high heterogeneity and uneven gender ratio), imprecision (small sample size) and risk of bias (unclear blinding). This is illustrated in Figure 14.

## 4 Discussion

### 4.1 Summary of main findings

This study evaluated the efficacy and safety of Tanshinone capsules in treating acne vulgaris. A total of 28 trials involving 2,969 patients with acne vulgaris were included. The meta-analysis results demonstrated that Tanshinone capsules had a significant impact on reducing acne recurrence rates, lowering GAGS scores for skin lesions, and decreasing sebum secretion rates, suggesting that Tanshinone capsules can offer a significant and long-lasting improvement in acne lesions and associated symptoms. Furthermore, analysis of inflammatory factor levels indicated that Tanshinone capsules significantly reduced TNF-α, IL-4, IL-6, and IL-8 levels. Suggesting that Tanshinone capsules can effectively alleviate inflammation and prevent the progression of inflammatory conditions. Given the close relationship between acne onset and patients’ hormone levels, we also conducted a meta-analysis of hormone levels, the results showed that Tanshinone capsules effectively lowered testosterone levels, contributing to acne treatment. The effects of Tanshinone capsules on acne are attributable to multi-factors. However, although previous studies have reported that Tanshinone exhibits both anti-androgenic effects and estrogen-like activity, the meta-analysis did not find any significant differences in LH, FSH, or E_2_ levels. In terms of safety, none of the included studies reported severe adverse events, and the meta-analysis results showed that Tanshinone capsules were associated with fewer adverse events compared to control groups. In summary, Tanshinone capsules effectively improve acne lesions, reduce excessive sebum secretion, regulate inflammatory markers and testosterone levels, and demonstrate long-lasting efficacy with a favorable safety profile.

### 4.2 Applicability of evidence

Tan ⅡA and CPT, the main active ingredients in Tanshinone capsules, show antibacterial activity against acne-related pathogenic microorganisms *in vitro* studies (Zhu et al., 2022; Li and Zhou, 2018). *C. acnes* activates the NF-κB pathway through TLR2, resulting in elevated levels of IL-1β, IL-6,IL-8, and TNF-α (Cong et al., 2019). Tanshinone can reduce the levels of IL-8, IL-6, IL-1β, and TNF-α in acne model rats, as profiled by lipidomics, with the mechanism possibly related to sphingolipid and glycerophospholipid metabolism pathways (Chen et al., 2021). Tan ⅡA treatment inhibits the TLR2/NF-κB pathway and suppress the expression of inflammatory cytokines IL-1β, IL-8, and TNF-α (Li and Zhou, 2018). CPT contributes to reducing the levels of inflammatory cytokines IL-1β, IL-6, IL-8, and TNF-α in acne model rats. This effect may be related to downregulating the expression of keratin, inhibiting glycolysis/gluconeogenesis and histidine metabolism, modulating lipid metabolism and altering sebum production, as well as downregulating the IL-17 signaling pathway, based on proteomics and metabolomics (Zhu et al., 2021). CPT helps restore the structure of skin microbiota, improve lipid metabolite composition and concentration, and negatively regulate the glycolysis pathway, thereby inhibiting excessive keratinocyte proliferation and reducing acne inflammation. (Zhu et al., 2022). Tan ⅡA and CPT can inhibit sebocyte proliferation and lipid synthesis, as well as downregulate AR expression (Ju et al., 2005). IL-4 expression is increased in acne hypertrophic scars, which is significantly importance for the prognosis of acne vulgaris (Yang J. H. et al., 2018). However, due to the unclear mechanism of Tanshinone’s effect on IL-4 during the course of acne, these results should be interpreted with caution. Furthermore, other studies have found that Tanshinones exhibit both anti-androgenic and estrogen-like activity (Zhang et al., 2019; Siddique et al., 2012).

Overall, Tanshinones can intervene in the growth of pathogenic microorganisms, lower inflammatory cytokine levels to alleviate inflammatory responses, suppress sebaceous gland hyperplasia and excessive lipid secretion, and regulate hormone levels, thereby treating acne.

### 4.3 Secondary findings

We assessed the impact of course of treatment and intervention methods in the experimental groups on the recurrence rate and levels of inflammatory factors (excluding IL-4), which are relevant to clinical practice. We found that in different treatment duration subgroups, Tanshinone capsules consistently reduced recurrence rates and TNF-α levels. This effect was also observed when Tanshinone capsules were used as a monotherapy or as an adjunctive therapy in combination treatments. Notably, results of the subgroup analyses were not entirely consistent. For IL-6 and IL-8 levels, Tanshinone capsules used as monotherapy were less effective than conventional treatments. Additionally, subgroup analysis indicated that when the treatment duration was 6 weeks or less, there was no significant difference between the treatment and control groups in IL-6 and IL-8 levels. We speculate that these discrepancies may be due to the limited number of included studies, as these subgroups typically consist of only one or two studies. Therefore, these findings should be interpreted with caution.

The sensitivity analysis showed that most results were stable. However, after excluding a single study, we observed instances where the outcomes for IL-6, IL-8, T, FSH, and sebum secretion rate exceeded the original 95% confidence interval, lost statistical significance, or exhibited increased heterogeneity. These findings indicate potential result instability, which should be interpreted with caution.

### 4.4 Limitations

Some limitations of this study need to be addressed. First, since all the included studies were conducted in China, the generalizability of the results to other ethnicities may be limited. Further research is needed to validate these findings across different ethnic groups. Second, the included studies often lack a randomized controlled trial framework and provide insufficient details on patient stratification criteria, leading to an increased risk of bias that reduces the credibility of the findings. Therefore, the conclusions should be interpreted with caution. Third, since the mechanism of Tanshinone’s effect on inflammatory cytokines in acne has not been fully elucidated, the related results should be interpreted with caution. Fourth, the maximum treatment duration was 3 months (only 1 study). Due to the limited number of studies and follow-up data, the long-term clinical benefits and potential risks associated with extended treatment durations remain unclear. Finally, due to the varying criteria used to evaluate adverse events and interventions across studies, the results regarding AEs should be interpreted with caution.

### 4.5 Implications for practice

The findings of our study indicate that Tanshinone capsules have great potential for acne treatment. This medication could be considered as a complementary medicine and deserves further exploration. In terms of future clinical study designs, large-sample, multicenter, long-term RCTs should be conducted, strictly adhering to the Consolidated Standards of Reporting Trials (CONSORT) guidelines. At the same time, the quality could be improved by standardizing study protocols, unifying diagnostic criteria, enhancing randomization, allocation concealment, and blinding, as well as appropriately calculating sample sizes. In addition, it is essential to implement detailed long-term toxicity monitoring, create standardized patient follow-up protocols, select objective and scientific outcome indicators, and evaluate the impact on acne-related quality of life.

Furthermore, conducting comprehensive molecular and experimental pharmacological analysis, identifying potential biomarkers for predicting treatment response, and investigating the molecular mechanisms of Tan ⅡA and CPT in relation to their respective targets will enhance our understanding of the molecular characterization of Tanshinone. Simultaneously, applying advanced imaging and molecular tracking techniques, developing computational models to predict treatment response, and validating molecular mechanisms in clinical research will provide better guidance for clinical practice.

## 5 Conclusion

This study confirmed that Tanshinone capsules can alleviate acne lesions, reduce sebum secretion, lower recurrence rates, and regulate inflammatory factors and hormone levels. However, due to the low quality of evidence in the included studies, further well-designed, multicenter studies with large sample sizes and high methodological rigor are needed to validate these findings.

## Data Availability

The original contributions presented in the study are included in the article/Supplementary Material, further inquiries can be directed to the corresponding authors.
