# Supplementary material for: Efficacy and safety of Tanshinone capsule in Acne vulgaris: a systematic review and meta-analysis
Source: Front Pharmacol. 2025 Mar 31;16:1520039. doi: 10.3389/fphar.2025.1520039 (PMC11994734; doi:10.3389/fphar.2025.1520039)
Supplement: Supplementary file 1 [file DataSheet1.docx]

**Supplementary Materials**

**catalogue**

**[1 Supplementary Tables 2](#_Toc20578)**

[2.1 Table S1 PRISMA checklist of the meta-analysis. 2](#_Toc2801)

[2.2 Table S2 Reasons for exclusion of studies after full text review. 6](#_Toc2928)

[2.3 Table S3 Botanical drugs included and traditional effects of Tanshinone Capsule 13](#_Toc23968)

[2.4 Table S4 Search strategies. 13](#_Toc32631)

**[2 Supplementary Figures 16](#_Toc22129)**

[2.1 Supplementary Figure S1. Sensitivity analysis 16](#_Toc19188)

# Supplementary Tables

**2.1 Table S1 PRISMA checklist of the meta-analysis.**

| **Section and Topic** | **Item #** | **Checklist item** | **Location where item is reported** |
| --- | --- | --- | --- |
| **TITLE** | | |  |
| Title | 1 | Identify the report as a systematic review. | Title |
| **ABSTRACT** | | |  |
| Abstract | 2 | See the PRISMA 2020 for Abstracts checklist. | Abstracts |
| **INTRODUCTION** | | |  |
| Rationale | 3 | Describe the rationale for the review in the context of existing knowledge. | Introduction |
| Objectives | 4 | Provide an explicit statement of the objective(s) or question(s) the review addresses. | Introduction |
| **METHODS** | | |  |
| Eligibility criteria | 5 | Specify the inclusion and exclusion criteria for the review and how studies were grouped for the syntheses. | Inclusion criteria |
| Information sources | 6 | Specify all databases, registers, websites, organisations, reference lists and other sources searched or consulted to identify studies. Specify the date when each source was last searched or consulted. | Literature search |
| Search strategy | 7 | Present the full search strategies for all databases, registers and websites, including any filters and limits used. | Table S4 |
| Selection process | 8 | Specify the methods used to decide whether a study met the inclusion criteria of the review, including how many reviewers screened each record and each report retrieved, whether they worked independently, and if applicable, details of automation tools used in the process. | Literature search |
| Data collection process | 9 | Specify the methods used to collect data from reports, including how many reviewers collected data from each report, whether they worked independently, any processes for obtaining or confirming data from study investigators, and if applicable, details of automation tools used in the process. | Literature screening and risk of bias |
| Data items | 10a | List and define all outcomes for which data were sought. Specify whether all results that were compatible with each outcome domain in each study were sought (e.g. for all measures, time points, analyses), and if not, the methods used to decide which results to collect. | Outcome measures and Exclusion criteria |
|  | 10b | List and define all other variables for which data were sought (e.g. participant and intervention characteristics, funding sources). Describe any assumptions made about any missing or unclear information. | TABLE 1 |
| Study risk of bias assessment | 11 | Specify the methods used to assess risk of bias in the included studies, including details of the tool(s) used, how many reviewers assessed each study and whether they worked independently, and if applicable, details of automation tools used in the process. | Literature screening and risk of bias |
| Effect measures | 12 | Specify for each outcome the effect measure(s) (e.g. risk ratio, mean difference) used in the synthesis or presentation of results. | Data analysis and GRADE assessment |
| Synthesis methods | 13a | Describe the processes used to decide which studies were eligible for each synthesis (e.g. tabulating the study intervention characteristics and comparing against the planned groups for each synthesis (item #5)). | Literature screening and risk of bias |
|  | 13b | Describe any methods required to prepare the data for presentation or synthesis, such as handling of missing summary statistics, or data conversions. | Literature screening and risk of bias |
|  | 13c | Describe any methods used to tabulate or visually display results of individual studies and syntheses. | Literature screening and risk of bias |
|  | 13d | Describe any methods used to synthesize results and provide a rationale for the choice(s). If meta-analysis was performed, describe the model(s), method(s) to identify the presence and extent of statistical heterogeneity, and software package(s) used. | Literature screening and risk of bias |
|  | 13e | Describe any methods used to explore possible causes of heterogeneity among study results (e.g. subgroup analysis, meta-regression). | Literature screening and risk of bias |
|  | 13f | Describe any sensitivity analyses conducted to assess robustness of the synthesized results. | Literature screening and risk of bias |
| Reporting bias assessment | 14 | Describe any methods used to assess risk of bias due to missing results in a synthesis (arising from reporting biases). | Data analysis and GRADE assessment |
| Certainty assessment | 15 | Describe any methods used to assess certainty (or confidence) in the body of evidence for an outcome. | Data analysis and GRADE assessment |
| **RESULTS** | | |  |
| Study selection | 16a | Describe the results of the search and selection process, from the number of records identified in the search to the number of studies included in the review, ideally using a flow diagram. | Literature screening and FIGURE 1 |
|  | 16b | Cite studies that might appear to meet the inclusion criteria, but which were excluded, and explain why they were excluded. | Table S2 |
| Study characteristics | 17 | Cite each included study and present its characteristics. | Characteristics of included studies and TABLE 1 |
| Risk of bias in studies | 18 | Present assessments of risk of bias for each included study. | Risk of bias assessment and FIGURE 2 |
| Results of individual studies | 19 | For all outcomes, present, for each study: (a) summary statistics for each group (where appropriate) and (b) an effect estimate and its precision (e.g. confidence/credible interval), ideally using structured tables or plots. | Effectiveness and safety of Tanshinone capsules and FIGURE3-12 |
| Results of syntheses | 20a | For each synthesis, briefly summarise the characteristics and risk of bias among contributing studies. | Effectiveness and safety of Tanshinone capsules and FIGURE3-12 |
|  | 20b | Present results of all statistical syntheses conducted. If meta-analysis was done, present for each the summary estimate and its precision (e.g. confidence/credible interval) and measures of statistical heterogeneity. If comparing groups, describe the direction of the effect. | Effectiveness and safety of Tanshinone capsules and FIGURE3-12 |
|  | 20c | Present results of all investigations of possible causes of heterogeneity among study results. | Effectiveness and safety of Tanshinone capsules and FIGURE3-12 |
|  | 20d | Present results of all sensitivity analyses conducted to assess the robustness of the synthesized results. | Figure S1 |
| Reporting biases | 21 | Present assessments of risk of bias due to missing results (arising from reporting biases) for each synthesis assessed. | FIGURE13 |
| Certainty of evidence | 22 | Present assessments of certainty (or confidence) in the body of evidence for each outcome assessed. | FIGURE14 |
| **DISCUSSION** | | |  |
| Discussion | 23a | Provide a general interpretation of the results in the context of other evidence. | Applicability of evidence |
|  | 23b | Discuss any limitations of the evidence included in the review. | Limitations |
|  | 23c | Discuss any limitations of the review processes used. | Limitations |
|  | 23d | Discuss implications of the results for practice, policy, and future research. | Implications for practice |
| **OTHER INFORMATION** | | |  |
| Registration and protocol | 24a | Provide registration information for the review, including register name and registration number, or state that the review was not registered. | Systematic Review Registration |
|  | 24b | Indicate where the review protocol can be accessed, or state that a protocol was not prepared. | Systematic Review Registration |
|  | 24c | Describe and explain any amendments to information provided at registration or in the protocol. | Study registration and ethics statements |
| Support | 25 | Describe sources of financial or non-financial support for the review, and the role of the funders or sponsors in the review. | Funding |
| Competing interests | 26 | Declare any competing interests of review authors. | Conflict of interest |
| Availability of data, code and other materials | 27 | Report which of the following are publicly available and where they can be found: template data collection forms; data extracted from included studies; data used for all analyses; analytic code; any other materials used in the review. | Data availability statement |

**2.2 Table S2 Reasons for exclusion of studies after full text review.**

| Exclusion reasons （n） | Study ID | Title of excluded studies |
| --- | --- | --- |
| The intervention did not meet the inclusion criteria (n = 23) | Wang YF 2018 | Clinical Observation on Tanshinone Combined with Red Light and Drug Mask in Treating Mild-moderate Acne |
|  | Pei C 2017 | Observation on the Effect of Adapalene Combined with Tanshinone Capsules in the Treatment of mild-moderate Acne Vulgaris |
|  | Zhang YZ 2017 | Clinical Efficacy of Tanshinone Capsules Combined with Ciprofloxacin Hydrochloride Gel in the Treatment of 60 Children with Facial Acne |
|  | Yuan LL 2015 | Comparative Study on the Clinical Efficacy of Red and Blue Light Combined with Medication in the Treatment of Acne Vulgaris |
|  | Wang YH 2014 | Efficacy Observation of Spironolactone and Tanshinone Capsules in the Treatment of Female Late-Onset Acne Vulgaris |
|  | Liu L 2013 | Danshentong Capsules Combined with Acne Extrusion and Drug Inverted Membrane Surgery for Acne Vulgaris |
|  | Lan XA 2013 | Comparative Observation on the Efficacy of Tanshinone and Tetracycline in the Treatment of Acne Vulgaris |
|  | Li Y 2013 | Observation on the Efficacy of Doxycycline Combined with Tanshinone in the Treatment of Acne |
|  | Liu J 2012 | Observation on the Efficacy of Tanshinone Capsules Combined with Fusidic Acid Cream in the Treatment of Acne Vulgaris |
|  | Deng ZJ 2011 | Efficacy Observation of Tanshinone Capsule Combined with 5% Benzoyl Peroxide Gel in the Treatment of Acne Vulgaris |
|  | Pu J 2011 | Clinical observation of Tanshinone in combination with Doxycycline on treatment Of inflammatory acne |
|  | Yu ZJ 2009 | Efficiency of Tanshinone on Treating Late-onset Female Patients with Acne and the Affection on the Serum Levels of Sex Hormone |
|  | Liu DH 2008 | Observation of the Efficacy of Tanshinone Capsules Combined with Cuochuangwang in the Treatment of Acne |
|  | Lin LY 2008 | Low-Dose Viaminate Combined with Tanshinone for the Treatment of Acne |
|  | Xiao GL 2008 | Evaluation of the Efficacy of Isotretinoin Capsules Combined with Tanshinone Capsules in the Treatment of Nodular cystic Acne |
|  | Chen PR 2008 | Combination of Traditional Chinese Medicine and Western Medicine in the Treatment of 40 Cases of Acne Vulgaris |
|  | Xu JY 2008 | Clinical Observation of the Efficacy of a Self-Formulated Xiaocuotang Decoction in the Treatment of Acne |
|  | Chen Y 2007 | Combination of Narrow-Band Blue Light and Red Light Therapy with Medication for the Treatment of Acne Vulgaris |
|  | Ou BS 2006 | Clinical Observation of the Efficacy of Tanshinone Capsules Combined with Bimixin Gel in the Treatment of Post-Pubertal Acne in Women |
|  | Feng AP 2006 | Clinical Observation of the Efficacy of Tanshinone Capsules Combined with Sanrui Capsules in the Treatment of Acne Vulgaris |
|  | Feng MJ 2006 | Treatment of 89 Cases of Acne Vulgaris with Tanshinone Combined with Retinoic Acid Cream |
|  | Zhang XB 2006 | Efficacy Observation of Isotretinoin Capsules Combined with Tanshinone Capsules in the Treatment of Acne Vulgaris |
|  | Wang ZY 2004 | Study on Serum Hormone Levels in Acne Vulgaris Patients and the Effect of Tanshinone |
| The design did not meet the inclusion criteria (n = 13 ) | Zhu LY 2021 | Observation of the Clinical Efficacy of Tanshinone Combined with Fusidic Acid in the Treatment of Inflammatory Acne |
|  | Chen Y 2021 | Study on the Therapeutic Effect of Tanshinone Capsules Combined with Red-blue Light Irradiation on Acne Vulgaris in Yangjiang Area |
|  | Shao K 2020 | The Effect of Tanshinone combined with Isotretinoin on the Levels of IL-8 and TNF-α in Acne Patients |
|  | Zhu Z 2019 | Therapeutic Effect of Tanshinone Combined with Adapalene Gel in the Treatment of Mild to Moderate Acne |
|  | Zhang JF 2018 | Clinical Efficacy Analysis of Intense pulsed Light Automatic Pulse Combined with Tanshinone in the Treatment of Acne |
|  | Tan QW 2017 | Observation of the Therapeutic Effect of Tanshinone Capsules Combined with Carnatiao-86 Photon Therapy Device on Acne |
|  | Liu W 2016 | The Effect of Tanshinone Capsules Combined with Spironolactone Treatment on Sex Hormone Levels in Acne Patients |
|  | Yin XW 2012 | Clinical efficacy of red flea Treatment of acne |
|  | Kong XH 2010 | Clinical Observation on the Treatment of 299 Cases of Acne with Tanshinone |
|  | Kong FL 2009 | Clinical Observation of Tanshinone Combined with Clindamycin Gel in the Treatment of Acne Vulgaris |
|  | Peng JN 2008 | Observation of the Therapeutic Effect of Tanshinone on 40 Cases of Female Post Adolescent Acne |
|  | Liu XH 2007 | Observation of the Therapeutic Effect of Tanshinone Capsules on 64 cases of Acne Vulgaris |
|  | Wu GL 2005 | Analysis of 120 cases of acne treated with low-dose tanshinone |
| Not retrieved (n = 7) | Zhu Y 2022 | The efficacy of Tanshinone capsules combined with Minocycline Hydrochloride capsules in treating Acne Vulgaris |
|  | Liang J 2013 | Observation of the efficacy of Tanshinone combined with red and blue light therapy in treating Acne Vulgaris |
|  | Chen K 2013 | Observation of the efficacy of Doxycycline Hydrochloride combined with Tanshinone in treating moderate to severe Acne |
|  | Wang YH 2005 | Observation of the clinical efficacy of Tanshinone in treating Acne Vulgaris |
|  | Liu YJ 2005 | Observation of the clinical efficacy of Tanshinone in treating Acne Vulgaris |
|  | Wang JY 2004 | Observation of the efficacy of Tanshinone combined with Minocycline in treating 36 cases of Acne |
|  | Jiang MB 2004 | Observation of the efficacy of Tanshinone in treating 78 cases of Acne |
| The outcome did not meet the inclusion criteria (n = 57) | Wang J 2023 | Observing the Efficacy of Tanshinone on Mild to Moderate Acne Vulgaris |
|  | Zheng QH 2021 | Analysis of Tanshinone Capsules Combined with Isotretinoin Erythromycin Gel in the Treatment of Acne |
|  | Lei GQ 2019 | The Clinical Efficacy of 540 Nm Intense Pulsed Light Combined with Tanshinone Capsule in the Treatment of Facial Acne |
|  | Li XG 2019 | The Study on Clinical Efficacy of 5-aminolevulinic Acid Photodynamic Therapy in the Treatment of Moderate to Severe Acne |
|  | Yang HJ 2019 | Clinical Observation of the Efficacy of Tanshinone Capsules in Acne Treatment |
|  | He LJ 2019 | Observation of the Therapeutic Effect of Isotretinoin Capsules Combined with Tanshinone Capsules in the Treatment of Moderate to Severe Acne |
|  | Yang XL 2018 | Experience of Treating Acne with Tanshinone Capsules Combined with Fusidic Acid Cream |
|  | Lu ZL 2018 | Clinical Observation of the Efficacy of Tanshinone Combined with Fusidic Acid in the Treatment of Inflammatory Acne |
|  | Chen SN 2018 | Clinical Observation of Tanshinone Combined with Isotretinoin Capsules in the Treatment of Acne |
|  | Yang GX 2018 | Therapeutic effect of doxycycline hydrochloride combined with tanshinone on mild acne vulgaris |
|  | Zhao WH 2018 | Observation of the Clinical Efficacy of Isotretinoin Capsules Combined with Tanshinone in the Treatment of Moderate to Severe Acne |
|  | Xie SH 2017 | Observation on the Therapeutic Effect of 5-aminolevulinic Acid Gel-photodynamic Therapy Combined with Tanshinone Capsules in the Treatment of Moderate Acne |
|  | Chen WD 2017 | Clinical Observation on Modified Baihu Decoction in Treatment of Acne |
|  | Li TJ 2017 | Tanshinone Capsule Combined with Erythromycin Isotretinoin Gel in the Treatment of Mild to Moderate Acne Vulgaris |
|  | Li GY 2017 | Efficacy and Safety of Oral Tanshinone Combined with Minocycline in Treatment of Moderate to Severe Acne Vulgaris |
|  | Wang J 2017 | Observation of the Effect of Tanshinone and Retinoic Acid Ointment in the Treatment of Acne |
|  | Du LW 2016 | Clinical Observation of Adapalene Combined with Tanshinone in the Treatment of 30 Cases of Mild to Moderate Acne |
|  | Zhou C 2016 | Evaluation of the efficacy and safety of tanshinone capsule combined with viaminate capsule and erythromycin isotretinoin gel in the treatment of facial acne |
|  | Zhang H 2016 | The Effects of Tanshinone Capsule Combined with Red-blue Light Therapy on Treatment of Acne |
|  | Zheng D 2016 | Observation and Analysis of Clinical Effect by Tanshinone and Clindamycin Palmitate Hydrochloride in the Treatment of 60 Adolescent Facial Acne Patients |
|  | Huo YL 2016 | Efficacy of Glycolic Acid combined with Tanshinone on Acne Vulgaris |
|  | Shan HD 2016 | Observation of the Therapeutic Effect of Isotretinoin on Moderate Acne |
|  | Du H 2015 | Clinical Research of Tanshinone Capsule Combined with Photodynamic Therapy in the Treatment of 31 Cases Severe Acne |
|  | Huo YL 2015 | Observation of the Therapeutic Effect of Alternating Red and Blue Light Irradiation Combined with Tanshinone on Facial Acne Vulgaris |
|  | Ma F 2015 | Observation on the Treatment of 30 Cases of Acne Vulgaris with Tretinoin Ointment |
|  | Sun YL 2014 | Clinical Research of Tanshinone Capsule Combined with Photodynamic Therapy in the Treatment of Severe Acne |
|  | Luo HY 2014 | Clinical Observation of 68 Cases of Mild to Moderate Acne Vulgaris Treated with Tanshinone Combined with Erythromycin Isotretinoin Gel |
|  | Wang SQ 2014 | Observation of the Therapeutic Effect of Strong Pulsed Light Combined with Tanshinone on Acne |
|  | Hao L 2014 | Observation on the Efficacy of Erythromycin Isotretinoin Gel Combined with Tanshinone Capsules in the Treatment of Mild to Moderate Acne |
|  | Zhou JF 2014 | Observation of the Therapeutic Effect of Isotretinoin Combined with Tanshinone in the Treatment of Acne |
|  | Mao HJ 2013 | Observation on the Efficacy of Tanshinone Capsules Combined with Tretinoin Cream in the Treatment of Acne Vulgaris |
|  | Yang ZG 2012 | Analysis of the Efficacy of Tanshinone Capsules Combined with Fusidic Acid Cream in the Treatment of Acne Vulgaris |
|  | Ma J 2012 | Observation on the Clinical Efficacy of Tanshinone Capsules and Omnilux Blue-red LED Phototherapy Combination in the Treatment of Acne |
|  | Wei JX 2012 | Clinical Observation of Tanshinone Capsule in Treatment of Acne |
|  | Yin WH 2011 | The interventionObservation on the Efficacy of Tanshinone Capsules Combined with Adapalene Gel in the Treatment of Acne Vulgaris does not conform |
|  | Zheng HY 2011 | Observation of the Therapeutic Effect of Tanshinone Capsules on Acne |
|  | Yang XP 2011 | Therapeutic Effect of Tanshinone Combined with Facial Mask and Red-blue Light on Acne |
|  | Lu RJ 2011 | Observation of the Therapeutic Effect of Tanshinone Combined with Western Medicine on Acne Vulgaris |
|  | Shen CH 2011 | Observation of the Therapeutic Effect of Narrowband Blue Light Combined with Tanshinone in the Treatment of Female Post Adolescent Acne |
|  | Chen H 2009 | Clinical Observation of Acne Vulgaris with Joint US-tanshinone Capsule and Cyclophilin |
|  | Yang AQ 2009 | Observation of the Clinical Efficacy of Tanshinone in Treating 47 Cases of Acne |
|  | He JX 2009 | Observation on the Effect of 0.1%Tazarotene Cream Combined with Tanshinone Capsules on Facial Acne Vulgaris |
|  | Li YZ 2008 | Observation of the Therapeutic Effect of Tanshinone capsules on 120 cases of acne vulgaris |
|  | Huang H 2008 | Observation of the Clinical Efficacy of Tanshinone Combined with Tretinoin Ointment in the Treatment of 52 Cases of Acne Vulgaris |
|  | Chen YP 2008 | Clinical Observation of Tanshinone Combined with Benzoyl Peroxide Gel in the Treatment of Acne Vulgaris |
|  | He CX 2008 | Observation of the Therapeutic Effect of Viaminate Capsules Combined with Tanshinone in the Treatment of Acne Vulgaris |
|  | Li JH 2007 | Clinical Effeet of Danshentong Combined With Topieal Retinoids in Aene Treatment |
|  | Chen H 2007 | Observation on effect in 66 cases of acne treated with Salviae Miltiorrhizae ketone Capsule |
|  | Lai XJ 2007 | The Therapeutic Effect of Tanshinone on Female Post Adolescent Acne and Impact on Sebum Secretion |
|  | Ji J 2007 | Observation of the Clinical Efficacy of Tanshinone in the Treatment of Acne Vulgaris |
|  | Yang KL 2007 | Observation of the Therapeutic Effect of Erythromycin Sustained-release Combined with Tanshinone in the Treatment of Menstrual Acne |
|  | Gao YL 2007 | Observation of the Therapeutic Effect of Tanshinone combined with Viaminate Capsules in the Treatment of Acne Vulgaris |
|  | Song T 2006 | Observation of Tanshinone in the Treatment of Acne Vulgaris and Impact on Sebum Secretion |
|  | Wang GJ 2006 | Observation of the Therapeutic Effect of the Combination of Viaminate Capsules and Tanshinone Capsules in the Treatment of Acne Vulgaris |
|  | Hu YE 2005 | Observation of the Therapeutic Effect of Tanshinone Capsules and Viaminate Capsules Combination Therapy on Acne Vulgaris |
|  | Liu XY 2004 | Clinical Observation of Adapalene Gel and Tanshinone CapsuleS in the Treatment of Acne (Report of 60 Cases) |
|  | Wu J 2004 | Observation of Efficiency of Tanshinone on Treating Patients with Acne Vulgaris |

**2.3 Table S3 Botanical drugs included and traditional effects of Tanshinone Capsule**

| Name | Source species | Main component | Properties | Merdians | Traditional efficacy |
| --- | --- | --- | --- | --- | --- |
| Tanshinone Capsule | Salvia miltiorrhiza | Cryptotanshinone, Tanshinone IIA | slightly cold, Bitter | Heart, Liver | invigorating the blood circulation, ntibacterial and anti-inflammatory |

**2.4 Table S4 Search strategies.**

| **#** | **Searches** |
| --- | --- |
| **Search strategies of CNKI (Searched from Jan.1, 2004 to Sept. 1, 2024 and found 471 literature)** | |
| **#1** | (SU%痤疮 OR SU%寻常痤疮 SU%粉刺 OR SU%肺风粉刺) AND(SU%丹参酮 OR SU%丹参酮胶囊) AND(SU%随机对照试验 OR SU%随机 OR SU%临床 OR SU%疗效) |
| **Search strategies of Wanfang (Searched from Jan.1, 2004 to Sept. 1, 2024 and found 512 literature)** | |
| **#1** | 主题:(寻常痤疮 OR 痤疮 OR 粉刺 OR 肺风粉刺) and 主题:(丹参酮 OR 丹参酮胶囊) and 主题:(随机对照试验 OR 随机 OR 疗效 临床 OR 疗效)) and 发表时间:2004-2024 |
| **Search strategies of VIP (Searched from Jan.1, 2004 to Sept. 1, 2024 and found 202 literature)** | |
| **#1** | M=(寻常痤疮 OR 痤疮 OR 粉刺 OR 肺风粉刺) AND M=(丹参酮 OR 丹参酮胶囊) AND M=(随机对照试验 OR 随机 OR 临床 OR 疗效) |
| **Search strategies of SinoMed (Searched from Jan.1, 2004 to Sept. 1, 2024 and found 340 literature)** | |
| **#1** | "寻常痤疮"[不加权:扩展] |
| **#2** | "痤疮"[常用字段:智能] OR "粉刺"[常用字段:智能] OR "肺风粉刺"[常用字段:智能] |
| **#3** | **#1 OR #2** |
| **#4** | "丹参酮"[不加权:扩展] |
| **#5** | "丹参酮胶囊"[常用字段:智能] |
| **#6** | **#4 OR #5** |
| **#7** | "随机对照试验"[不加权:扩展] |
| **#8** | "随机"[常用字段:智能] OR "临床"[常用字段:智能] OR "疗效"[常用字段:智能] |
| **#9** | **#7 OR #8** |
| **#10** | **#3 AND #6 AND #9 AND 2004-2024[日期]** |
| **Search strategies of PubMed (Searched from Jan.1, 2004 to Sept. 1, 2024 and found 4 literature)** | |
| **#1** | "Acne Vulgaris"[Mesh] |
| **#2** | acne[Title/Abstract] |
| **#3** | **#1 OR #2** |
| **#4** | "tanshinone" [Supplementary Concept] |
| **#5** | (((((tanshinone capsules[Title/Abstract])) OR (tanshinone capsule[Title/Abstract])) OR (danshentong capsules[Title/Abstract])) OR (danshentong capsule[Title/Abstract])) OR (danshentong[Title/Abstract]) |
| **#6** | **#4 OR #5** |
| **#7** | randomized controlled trial[Publication Type] OR randomized[Title/Abstract] OR placebo[Title/Abstract] OR (clinical[Title/Abstract]) OR (efficacy[Title/Abstract]) |
| **#8** | #3 AND #6 AND #7 |
| **Search strategies of EMbase (Searched from Jan.1, 2004 to Sept. 1, 2024 and found 10 literature)** | |
| **#1** | 'acne vulgaris'/exp OR 'acne vulgaris' |
| **#2** | 'acne vulgaris':ab,ti OR 'acne':ab,ti |
| **#3** | **#1 OR #2** |
| **#4** | 'tanshinone'/exp OR 'tanshinone' |
| **#5** | 'danshentong capsules':ab,ti OR 'danshentong capsule':ab,ti OR 'tanshinone capsules':ab,ti OR 'tanshinone capsule':ab,ti OR 'tanshinone':ab,ti OR 'danshentong':ab,ti |
| **#6** | **#4 OR #5** |
| **#7** | 'randomized controlled trial':ab,ti OR 'randomized':ab,ti OR 'placebo':ab,ti OR 'rct':ab,ti OR 'rcts':ab,ti OR 'clinical':ab,ti OR 'efficacy':ab,ti |
| **#8** | #3 AND #6 AND #7 |
| **Search strategies of Cochrane Library (Searched from Jan.1, 2004 to Sept. 1, 2024 and found 4 literature)** | |
| **#1** | MeSH: Acne vulgaris |
| **#2** | (Acne Vulgaris):ab,ti,kw OR (Acne):ab,ti,kw |
| **#3** | **#1 OR #2** |
| **#4** | (Tanshinone):ab,ti,kw OR (Tanshinone Capsules):ab,ti,kw OR (Tanshinone Capsule):ab,ti,kw OR (danshentong Capsules):ab,ti,kw OR (danshentong Capsule):ab,ti,kw OR (danshentong):ab,ti,kw |
| **#5** | (randomized controlled trial):ab,ti,kw OR (randomized):ab,ti,kw OR (placebo):ab,ti,kw OR (RCT):ab,ti,kw OR (RCTs):ab,ti,kw OR (clinical):ab,ti,kw OR (efficacy):ab,ti,kw |
| **#6** | #3 AND #4 AND #5 |

# Supplementary Figures

## **2.1** **Supplementary Figure S1. Sensitivity analysis**

**
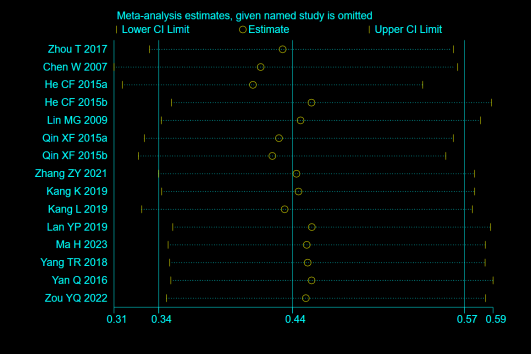

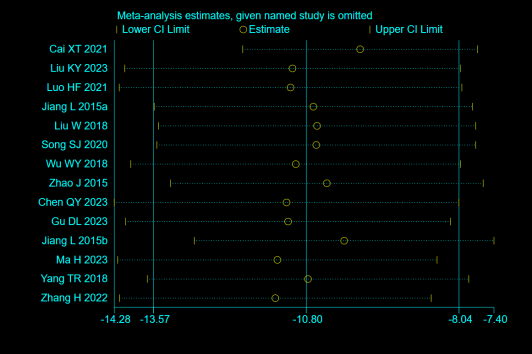
A B**

**
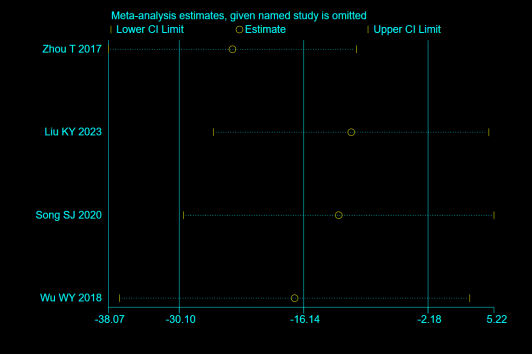

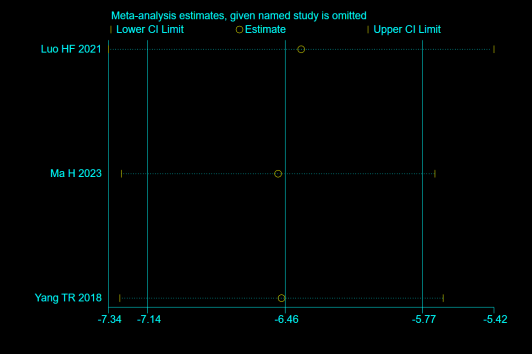
C D**

**
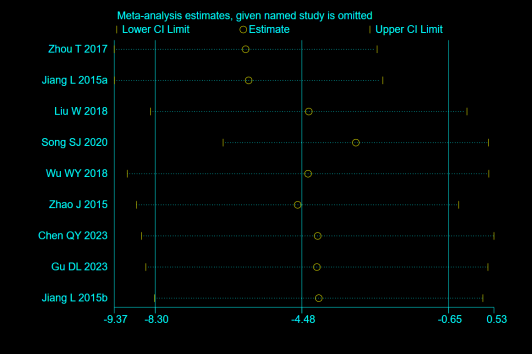

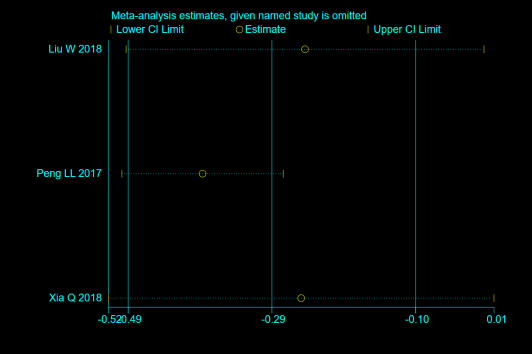
E F**

**
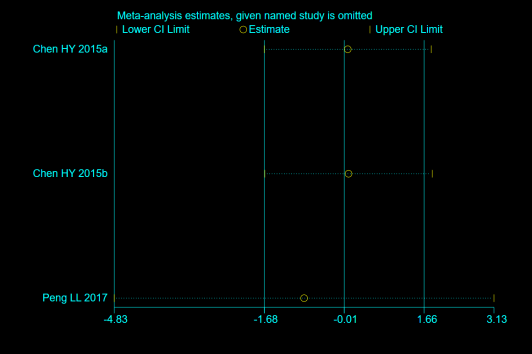

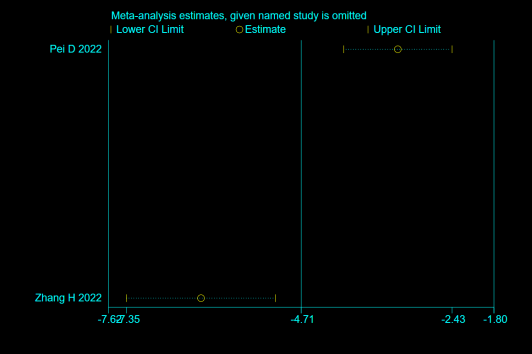
G H**

**
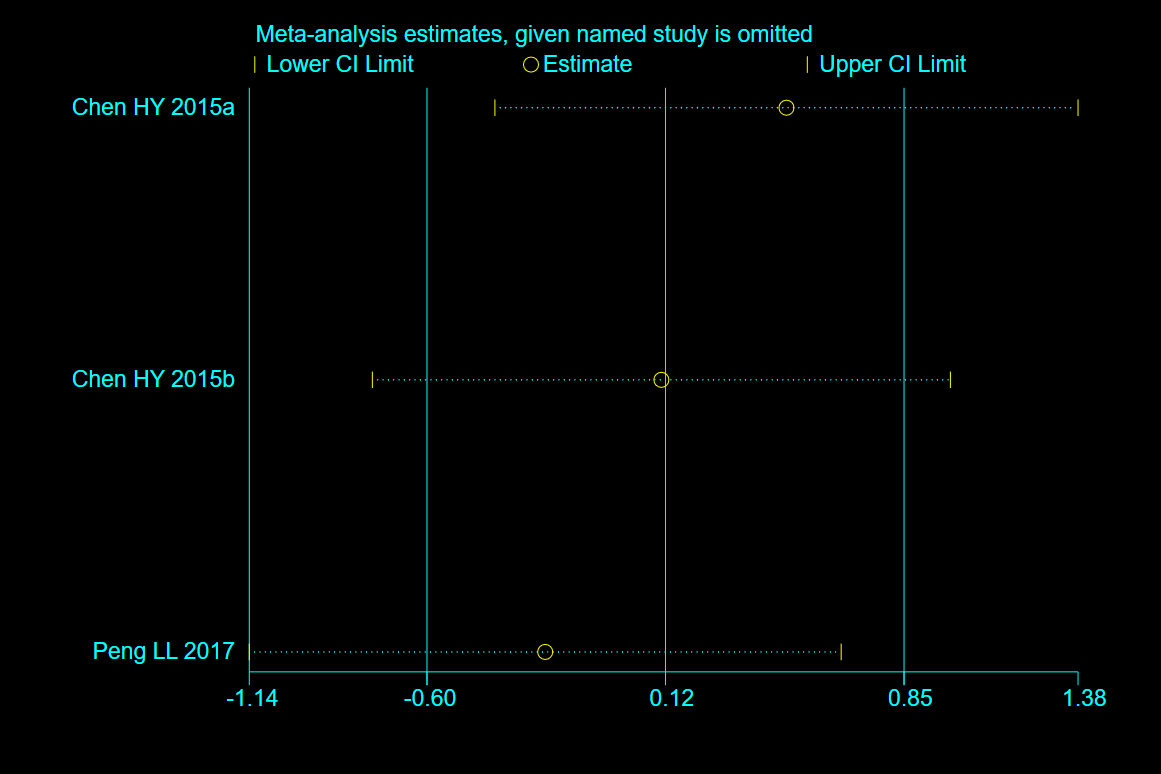

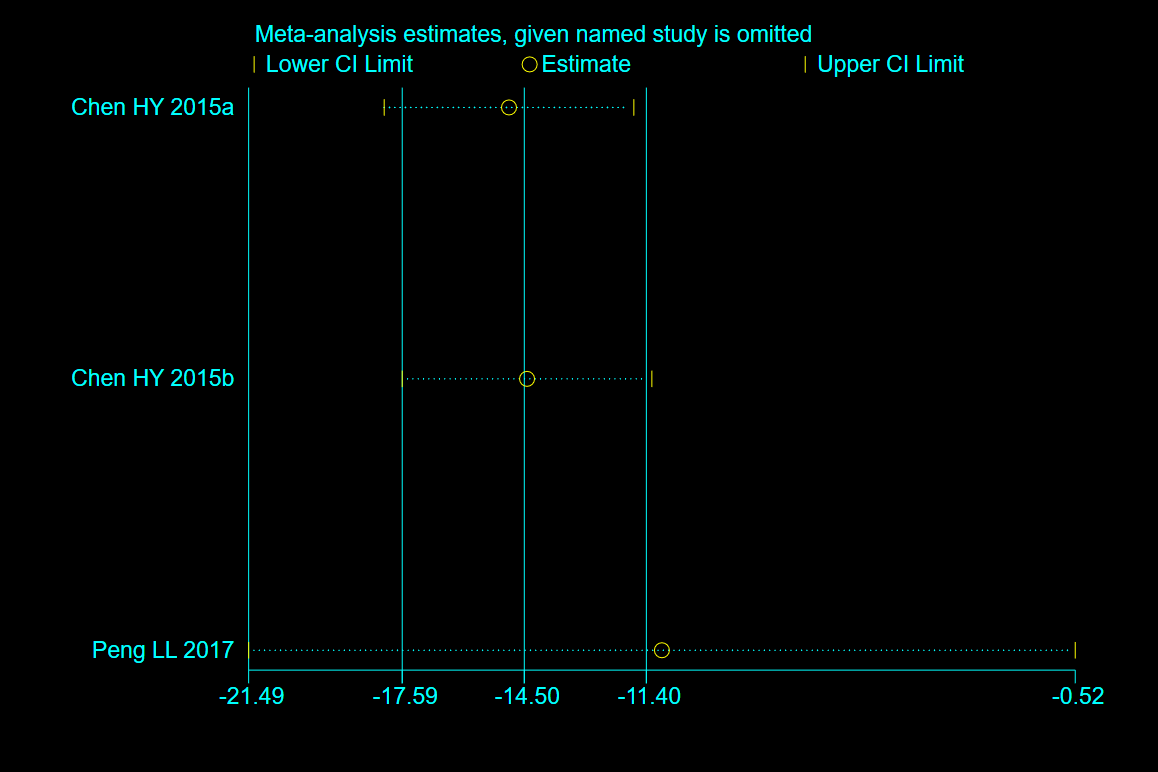
I J**

**
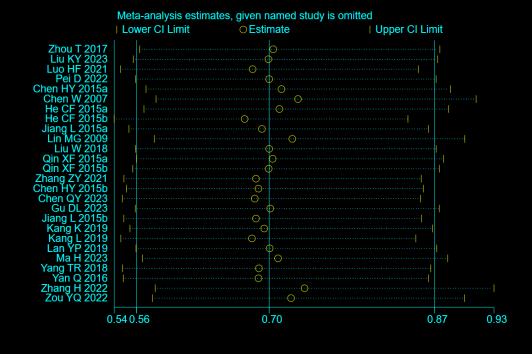

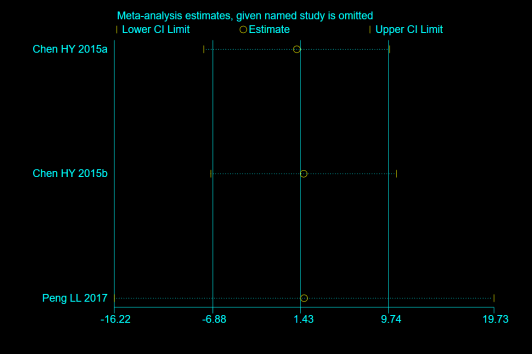
K L**

1. The relapse rate; **(B)** Levels of TNF-α; **(C)** Levels of IL-4; **(D)** Levels of IL-6; **(E)** Levels of IL-8; **(F)** Sebum secretion rate; **(G)** GAGS sores ; **(H)** Levels of LH; **(I)** Levels of FSH; **(J)** Levels of T; **(K)** Levels of E2; **(L)** AEs

**
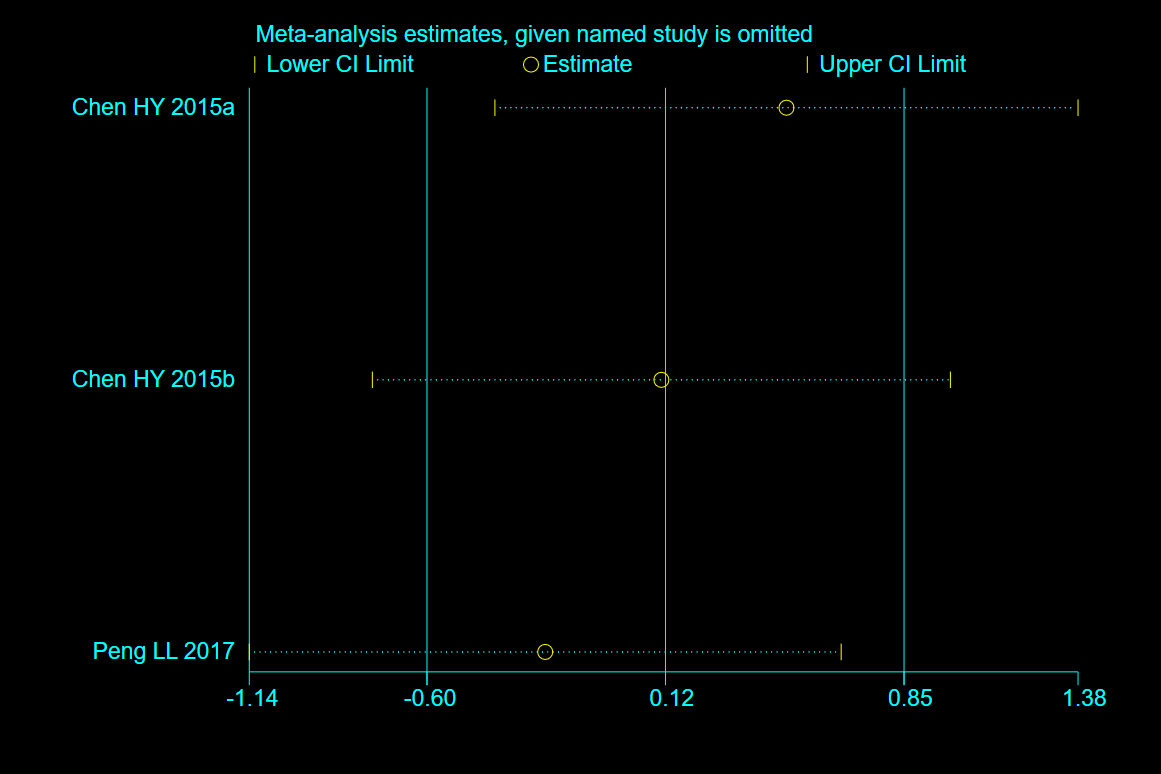

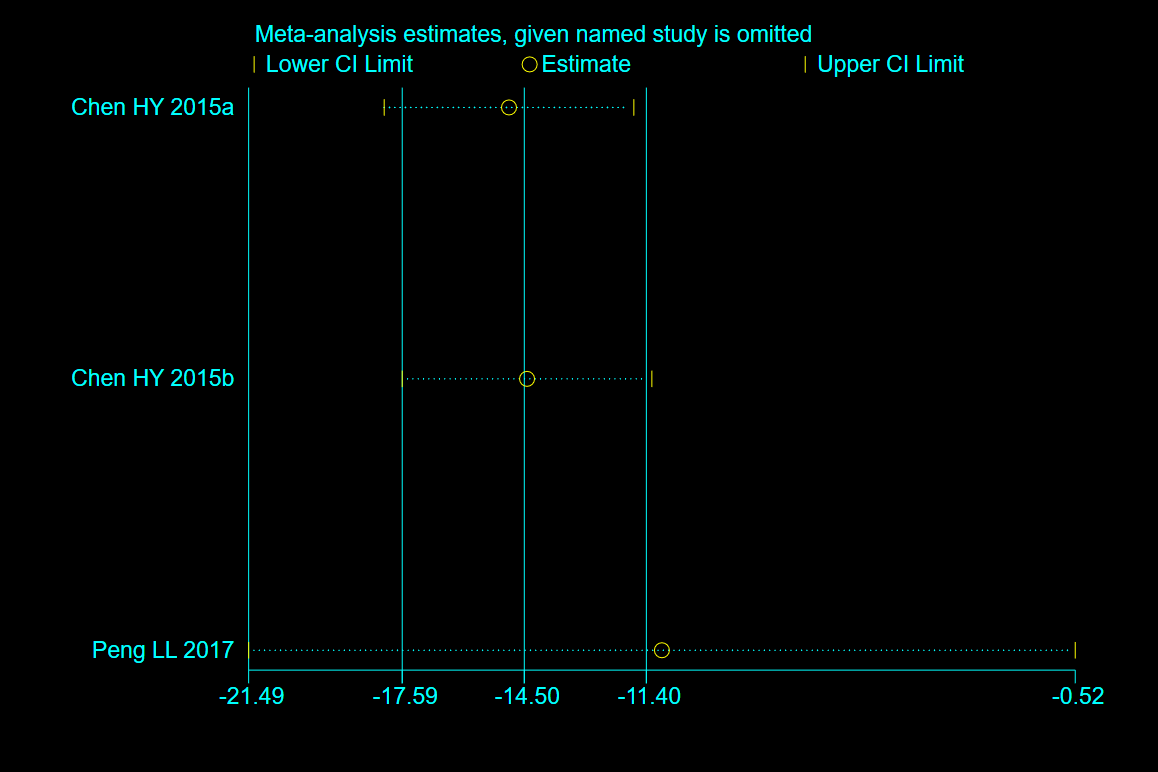
**
